# Microbial Communities on Plastic Polymers in the Mediterranean Sea

**DOI:** 10.3389/fmicb.2021.673553

**Published:** 2021-06-16

**Authors:** Annika Vaksmaa, Katrin Knittel, Alejandro Abdala Asbun, Maaike Goudriaan, Andreas Ellrott, Harry J. Witte, Ina Vollmer, Florian Meirer, Christian Lott, Miriam Weber, Julia C. Engelmann, Helge Niemann

**Affiliations:** ^1^Department of Marine Microbiology and Biogeochemistry, NIOZ Royal Netherlands Institute for Sea Research, Texel, Netherlands; ^2^Department of Molecular Ecology, Max Planck Institute for Marine Microbiology, Bremen, Germany; ^3^Inorganic Chemistry and Catalysis, Debye Institute for Nanomaterials Science, Utrecht University, Utrecht, Netherlands; ^4^HYDRA Marine Sciences GmbH, Bühl, Germany; ^5^Department of Earth Sciences, Utrecht University, Utrecht, Netherlands

**Keywords:** plastic polymer, biofilms, microbial community, hydrocarbon degrading bacteria, marine plastic debris

## Abstract

Plastic particles in the ocean are typically covered with microbial biofilms, but it remains unclear whether distinct microbial communities colonize different polymer types. In this study, we analyzed microbial communities forming biofilms on floating microplastics in a bay of the island of Elba in the Mediterranean Sea. Raman spectroscopy revealed that the plastic particles mainly comprised polyethylene (PE), polypropylene (PP), and polystyrene (PS) of which polyethylene and polypropylene particles were typically brittle and featured cracks. Fluorescence *in situ* hybridization and imaging by high-resolution microscopy revealed dense microbial biofilms on the polymer surfaces. Amplicon sequencing of the 16S rRNA gene showed that the bacterial communities on all plastic types consisted mainly of the orders Flavobacteriales, Rhodobacterales, Cytophagales, Rickettsiales, Alteromonadales, Chitinophagales, and Oceanospirillales. We found significant differences in the biofilm community composition on PE compared with PP and PS (on OTU and order level), which shows that different microbial communities colonize specific polymer types. Furthermore, the sequencing data also revealed a higher relative abundance of archaeal sequences on PS in comparison with PE or PP. We furthermore found a high occurrence, up to 17% of all sequences, of different hydrocarbon-degrading bacteria on all investigated plastic types. However, their functioning in the plastic-associated biofilm and potential role in plastic degradation needs further assessment.

## Introduction

Plastics as unmanaged waste are a great environmental concern (Xanthos and Walker, 2017; Wayman and Niemann, 2021). It is estimated that about 0.1% (Cozar et al., 2014) to up to 4.1% (Jambeck et al., 2015) of the globally produced plastics ultimately enter the ocean as so-called marine plastic debris (MPD). Since the 1950s, a total of ∼7,800 Mt of plastic has been produced, hence >350 Mt of MPD may have been released to the ocean (Wayman and Niemann, 2021). The size of MPD ranges from large items (macroplastics: >5 mm) to particles at the micrometer (microplastics: 1 μm–5 mm) and nanometer scale (nanoplastics). The breakdown of plastics in marine environments occurs due to shear stress and mechanical fragmentation (e.g., caused by wave action), weathering (e.g., photooxidation induced by UV radiation), or possibly due to microbial degradation. Weathering partially breaks carbon chains of the polymers and thus makes the plastics more bioavailable for microbes (Romera-Castillo et al., 2018).

Marine plastic debris accumulates in subtropical gyres, but substantial amounts of plastic get entrapped in closed or semiclosed water bodies such as the Mediterranean Sea (Cozar et al., 2015; van Sebille et al., 2015; Suaria et al., 2016). In fact, the Mediterranean Sea has been identified as the sixth hotspot for plastic accumulation besides the five subtropical gyres and is one of the most polluted marine regions in the world (Suaria et al., 2016). The composition of MPD in surface waters of the Mediterranean Sea varies (similar to other marine regions), but polyethylene (PE) and polypropylene (PP) appear to be the most abundant plastic types (Pedrotti et al., 2016; Suaria et al., 2016). Microplastics have been found floating in the Mediterranean surface waters (Cozar et al., 2015; Suaria et al., 2016; de Haan et al., 2019) as well as in coastal and deep-sea sediments (Vianello et al., 2013; Filgueiras et al., 2019; Kane et al., 2020), on beaches (Asensio-Montesinos et al., 2019; Piperagkas et al., 2019), and even within marine biota, such as fish (Giani et al., 2019), bivalves (Gonçalves et al., 2019), and corals (Savinelli et al., 2020).

Marine plastic debris introduces an artificial surface for microbial attachment in a habitat where free-living microbial cells would typically dominate. Living in a biofilm on MPD offers advantages over the free-living state for microbes: shelter against UV radiation, predation, and environmental changes, and it increases the odds of survival (de Carvalho, 2018; Vaksmaa et al., 2021). The colonization of bacteria on non-natural, submerged surfaces such as plastic can be initiated within hours (Siboni et al., 2007; Harrison et al., 2014). Plastic-biofilm aggregates may also become less buoyant, thus constituting one pathway of MPD removal from the ocean surface (Lobelle and Cunliffe, 2011; Kaiser et al., 2017). Plastics are rich in chemical energy, but it is unresolved if and how biofilm formation and microbes facilitate the degradation of MPD (Jacquin et al., 2019).

The microbial biofilm on the surface of different plastics is often composed of similar taxonomic groups, such as *Rhodobacteraceae*, *Flavobacteriaceae, Cyclobacteriaceae*, and *Alteromonadaceae* (Dang and Lovell, 2000; Zettler et al., 2013; Oberbeckmann et al., 2016; De Tender et al., 2017; Romera-Castillo et al., 2018; Miao et al., 2019; Schlundt et al., 2020). However, it remains a matter of debate if different polymers harbor and support differential microbial communities, if microbial communities on plastic differ from those on other inert surfaces and which microbes within these biofilms are potentially able to degrade the polymers (Zettler et al., 2013; Oberbeckmann et al., 2016; Frère et al., 2018; Kirstein et al., 2018; Pinto et al., 2019; Wright et al., 2020). Chemically, plastics are diverse, comprising hydrocarbon-like compounds with simple (e.g., polyolefins such as PE and PP) or more complex C–C skeletons [e.g., polystyrene (PS) featuring aromatic rings], which may also contain additional non-C heteroatoms, e.g., O and N [e.g., polyethylene terephthalate (PET) and polyamide (PA)]. Thus, for the degradation of plastics, microorganisms likely require an equally diverse set of enzymes to depolymerize/hydrolyze and oxidize the different plastic types. To date, several plastic-degrading microorganisms, including bacteria and fungi, have been isolated from various environments, although mostly from terrestrial systems (Gilan et al., 2004; Herrero Acero et al., 2011; Tanasupawat et al., 2016; Yoshida et al., 2016; Palm et al., 2019; Zhang et al., 2020). Typically, plastic degradation has been evaluated *in vitro* with plastic as the sole carbon source. *Rhodococcus ruber* was found to break down PE, PP, and PS (Gilan et al., 2004; Mor and Sivan, 2008; Gravouil et al., 2017), *Ideonella sakaiensis* degrades PET (Yoshida et al., 2016), and *Alcanivorax borkumensis* was shown to degrade low-density PE (LDPE) (Delacuvellerie et al., 2019). *Zalerion maritimum*, a marine fungal strain, was found to utilize PE (Paço et al., 2017), and *Aspergillus* spp. have been identified as potential high-density PE (HDPE) degraders (Sangeetha Devi et al., 2015). Although several *Vibrio* species have shown potential for plastic degradation (Raghul et al., 2014) and have often been found on plastic (Zettler et al., 2013; De Tender et al., 2015; Frère et al., 2018), they have mainly received attention as pathogens using plastic as means for hitchhiking to disperse (Kirstein et al., 2016; Silva et al., 2019). Seldomly have the enzymes involved in plastic degradation been identified (Jacquin et al., 2019). Strikingly, on genus and species level, plastic biofilms often constitute hydrocarbon or oil degraders. For example, *Arcobacter* and *Colwellia* have been found as dominant genera on LDPE (Harrison et al., 2014). *Alcanivorax*, *Marinobacter*, and *Arenibacter* genera became enriched on LDPE and PET in the Mediterranean Sea, and *Oleiphilus* and *Aestuariibacter* colonized PE an order of magnitude faster than glass surfaces (Erni-Cassola et al., 2020).

In this study, we collected floating marine plastic debris from a bay of the island of Elba (Mediterranean Sea). Our aims were (i) to analyze the microbial assemblages on single MPD particles, (ii) to identify taxa potentially relevant for plastic degradation with a specific focus on genera-containing hydrocarbon degraders, and (iii) to visualize the localization of specific microbial taxa on the plastic surface.

## Materials and Methods

### MPD Sampling and Fixation for Downstream Analysis

Plastic particles were collected in a small bay with a marina at the southern side of the Mediterranean Island Elba (Marina di Campo, 42.7427° N, 10.2384° E) on July 22, 2018. Sampling was carried out with a 150-μm handheld plankton net (Hydro-Bios, Germany). Particles were transferred into whirl packs prefilled with seawater from the sampling side and transported to a nearby laboratory. Here, plastic particles were sorted from other materials (e.g., floating insects or seaweed and wood debris) based on optical appearance (color, shape) using a stereomicroscope. After presorting, plastic particles were washed twice to remove the loosely attached biofilms and/or algae, by immersing single pieces into sterile-filtered (0.2 μm) seawater taken from the sampling site and gently shaking them. Half of the particles were placed into 2 ml vials prefilled with RNA later (Sigma-Aldrich, Germany) for downstream DNA extraction, and the second half were fixed for fluorescence *in situ* hybridization (FISH) and microscopy. For FISH, the microbial communities on single plastic pieces were fixed by placing plastic particles into 2 ml vials prefilled with 4% formaldehyde in 1× phosphate-buffered saline (PBS) at room temperature for 4 h. Subsequently, the particles were removed from the fixation solution and washed twice by gently shaking them in a Costar dish filled with 1× PBS. Finally, the samples were transferred into 2 ml cryovials filled with a freshly prepared 1× PBS:ethanol (1:1, vol/vol) solution. All samples were stored at −20°C.

### DNA Extraction and 16S rRNA Gene Amplicon Library Preparation

DNA from 40 single microplastic particles was extracted with the Powersoil DNA Isolation kit (Mo Bio Laboratories, Inc., Carlsbad, CA, United States) with minor modifications of the manufacturer’s recommendations: single particles were added to the PowerBead tubes directly. A bead-beating step (4.55 m/s for 30 s, three times, 10 s dwell time) was added to the protocol, replacing the original cell lysis step. The final elution was carried out with 30 μl of elution buffer. DNA concentrations were measured on a Qubit fluorometer with the Qubit dsDNA HS Assay Kit (Invitrogen, United States). The V4–V5 region of 16S rRNA genes were amplified in technical triplicate from extracted DNA using the universal primers 515F-Y (5′ GTGYCAGCMGCCGCGGTAA 3′) and 926R (5′ CCGYCAATTYMTTTRAGTTT 3′) (Parada et al., 2016). The forward and reverse primers were both barcoded with a unique 12 nucleotide Golay code. In short, the PCR reaction mix contained 25.5 μl PCR grade water, 10 μl 5× Phusion HF buffer, 4 μl dNTPs (2.5 mM), 2 μl BSA (920 mg/ml), 0.5 μl (2 U/μl) Phusion polymerase, and 3 μl of each forward and reverse primer (10 μM) and 2 μl of DNA. PCR was performed in a thermocycler with the following program: 98°C for 30 s followed by 35 cycles of 98°C for 30 s 50°C for 30 s, 72°C for 30 s, with a final elongation of 72°C for 7 min; thereafter, samples were kept at 4°C. All three technical PCR-replicated reactions were pooled in equimolar amounts and subjected to PCR purification by QIAquick PCR purification (QIAGEN, United States) according to manufacturer’s instructions, followed by 1% gel electrophoresis. The bands were visualized on a UV illuminator, excised from the gel and purified by QIAquick gel extraction (QIAGEN, United States). Illumina MiSeq 2x300 sequencing was carried out at the USEQ facility (Utrecht, Netherlands).

### Sequencing Data Analysis

The raw data were processed using the NIOZ in-house amplicon sequence analysis pipeline “Cascabel” (Abdala Asbun et al., 2020), according to the following specifications. First, quality on the reads was assessed with FastQC^1^, then paired-end reads were merged with PEAR v0.9.10 (Zhang et al., 2014) with a *p* value set to 0.05 (for the statistical test of a possible assembly) and a minimum overlap of 10 bases. Then, merged reads were demultiplexed using QIIME (Caporaso et al., 2010) v1.9 scripts extract_barcodes.py and split_libraries_fastq.py truncating reads at the first three consecutive bases with a *q* score below 20. Primers were removed using Cutadapt (Martin, 2011). Merged and trimmed reads longer than 357 nucleotides (nt) and shorter than 387 nt were retained for downstream analysis. The remaining reads were dereplicated with Vsearch (Rognes et al., 2016) and subsequently clustered by UCLUST (Edgar, 2010) at 97% of identity *via* pick_otus.py. Representative sequences for operational taxonomic units (OTU) were obtained *via* the pick_rep_set.py script using the longest method. The taxonomy assignment was made using Vsearch’s usearch_global method against the ARB Silva database (v132 Ref NR 99) (Quast et al., 2013) with a minimum identity of 70% according to the edit distance definition, accepting a maximum of three hits per query and retaining only the top hits. Taxonomy assignations were mapped to their lowest common ancestor with the stampa_merge.py script^2^ and the result was supplied to the make_otu_table.py script in order to build the OTU table and subsequent conversion to the biom format (McDonald et al., 2012). Finally, the biom table was summarized at different taxonomic levels, and singletons were filtered out by retaining OTUs with an abundance greater than 5 across any sample by using QIIME’s scripts summarize_taxa.py and filter_otus_from_otu_table.py, respectively. Despite using the universal primers 515F-Y and 926R and extracting the ∼400-bp fragment, described to amplify 16S rRNA gene of Bacteria and Archaea (Parada et al., 2016), we detected reads assigned to Eukarya (1.1–3.5%). Sequencing analysis and the commands used are described in detail in Supplementary Data Sheet 1. The sequences have been submitted to the Sequence Read Archive (SRA), bioproject PRJNA698954.

### Multivariate Analysis

Multivariate statistical analysis by using taxonomic classification on the order level and OTU level was carried out on the amplicon sequencing data. Firstly, OTUs with a total abundance fewer than 10 reads in total, OTUs present in only one sample, unassigned OTUs (631 reads), and secondly, OTUs classified as Eukarya (900 reads) were removed. In total, 5,335,114 reads were subjected to further analysis. Out of 40 samples, 38 (polymer identified) were used for statistical analysis using the PRIMER7 software package (version 7.0.13). To compensate for varying sequencing depth between samples, we first calculated the relative abundances of the total count of reads per sample (OTU and order level) and then applied square-root transformation on the relative abundances. With these data, Bray-Curtis similarity indices were calculated and visualized by non-metric multidimensional scaling (nMDS). Permutational multivariate analysis of variance (PERMANOVA) was applied to test for significant differences in the abundance of OTUs and orders between sample sets. A sample set was defined here as all microbial communities on single plastic pieces of one polymer type. Differences between individual groups were subjected to *post hoc* tests using permutational *t* test. Furthermore, PERMDISP was applied to test for homogeneity of dispersion between sample sets. We also applied similarity percentage analysis (SIMPER) to determine key OTUs/orders controlling community differences.

### Catalyzed Reporter Deposition Fluorescence *in situ* Hybridization and Preparation for Microscopy

The described procedures for staining cells and subsequent microscopy was nearly identical to the protocol described in Probandt et al. (2018). In brief, cells were permeabilized at 37°C with lysozyme (10 mg ml^–1^) for 60 min followed by achromopeptidase treatment (60 U ml^–1^) for 30 min. Endogenous peroxidases were inactivated in methanol containing 0.15% H_2_O_2_ for 20 min. Hybridization (3 h) using horseradish peroxidase-labeled probes and CARD step (1 h) was performed at 46°C as described previously, with the modification that hybridization was carried out in an eppendorf tube (Pernthaler et al., 2002a). During hybridization, vials were carefully inverted every 30 min to promote an efficient mixing of the hybridization buffer and plastic particles. After each step, the supernatant of the plastic particles was replaced three times with the solution needed for the following step. For multiple hybridizations, horseradish peroxidase from the first probe was inactivated in 0.01 M HCl with 0.15% H_2_O_2_ for 20 min. Tyramides (1.4 μg ml^–1^) were labeled with Alexa488, Alexa594, or Alexa647. For microscopy, plastic particles were placed in ibidi 8 well μ-Slide imaging chambers (Ibidi, Gräfelfing, Germany) and embedded in Citifluor/Vectashield (4:1) containing 4′,6-diamidino-2-phenylindole (DAPI) (0.5 μg ml^–1^) or Mowiol 4-88 (pH 7.5, adjusted with ascorbic acid) containing SYBR green I. Used probes and formamide concentrations are given in Supplementary Table 2.

### Image Acquisition Using Confocal Laser Scanning and Superresolution- Structured Illumination Microscopy

Microbial communities on plastic particles were visualized by confocal laser scanning microscopy (CLSM) using a Zeiss LSM 780 and by superresolution-structured illumination microscopy (SR-SIM) with Zeiss Elyra PS.1 (Zeiss, Jena, Germany). For both methods, a 63X/1.4 plan apochromatic oil objective was used as standard except for Figure 2B were a 10X/0.3 objective lens was used. For CLSM we excited DAPI, Alexa488, Alexa594, and Alexa647 using lasers with wavelengths of 405, 488, 561, and 633 nm. For SR-SIM, SYBR green I was excited with a 488-nm laser and detected using a 502- to 538-nm bandpass filter. The used SIM grating had a period of 42 μm. We imaged with three rotations and five phase shifts. The camera exposure time was set to 50 ms, with an EMCCD gain of 40, and four frames averaging. On all images, z-stacks for three-dimensional reconstruction were recorded. Image processing and visualization was done using the ZEN software package (Zeiss, Jena, Germany). The 3D video animation was done using the software Imaris 64X 8.0.2 (Bitplane, Zurich, Switzerland) after 3D blind deconvolution with AutoQuant X3 (Media Cybernetics, Inc., Rockville, MD, United States).

### Raman Spectrometry

After DNA extraction or FISH, the polymer composition of single MPDs was classified using Raman spectrometry using a InVia Raman microscope (Renishaw, United Kingdom) equipped with a 785-nm diode laser. MPDs were investigated through a 50× objective (0.75 NA, Leica, Germany) with a laser power of 50–100 mW, a grid with 1,200 lines mm^–1^ and integration time of 10–20 s per spectrum. For each sample, Raman spectra between wavenumbers of 200–3,000 cm^–1^ were recorded and matched against published values for Raman shifts (Clunies-Ross et al., 2016; Frère et al., 2016; Crawford and Quinn, 2017) to identify the polymer composition.

## Results

### Identity of Floating Plastics

**FIGURE 1:**
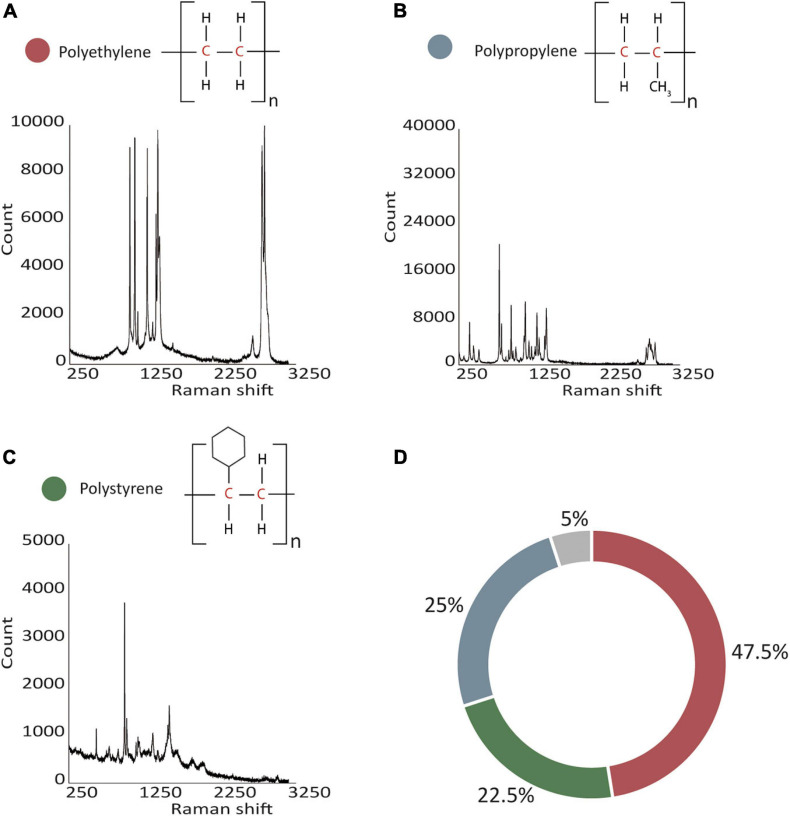
Classification and distribution of 40 plastic particles. **(A–C)** Identification of polymers was achieved with Raman spectroscopy, representative Raman spectra are shown. **(D)** Most particles were identified as PE followed by PP and PS, and 5% of particles remained unidentified (polyethylene in red, polypropylene in blue, polystyrene in green, and unidentified particles in gray).

Most plastic particles were irregularly shaped polygons (often resembling flat shards) or threads/fibers of various colors (mostly translucent, milky-whitish, blue, green, and orange, Supplementary Figure 1). Most particles also showed signs of mechanical stress, i.e., they featured cracks and fissures. Raman spectroscopy of 38 of the 40 particles yielded spectra allowing us to identify the polymer structure (two polymers could not be identified). The analyzed particles mainly consisted of PE (47.5%, 19 particles), PP (25%, 10 particles), and PS (22.5%, nine particles; Figure 1; see Raman spectra in the Supplementary Data Sheet 2).

### Microbial Community Structure on MPD

**FIGURE 2:**
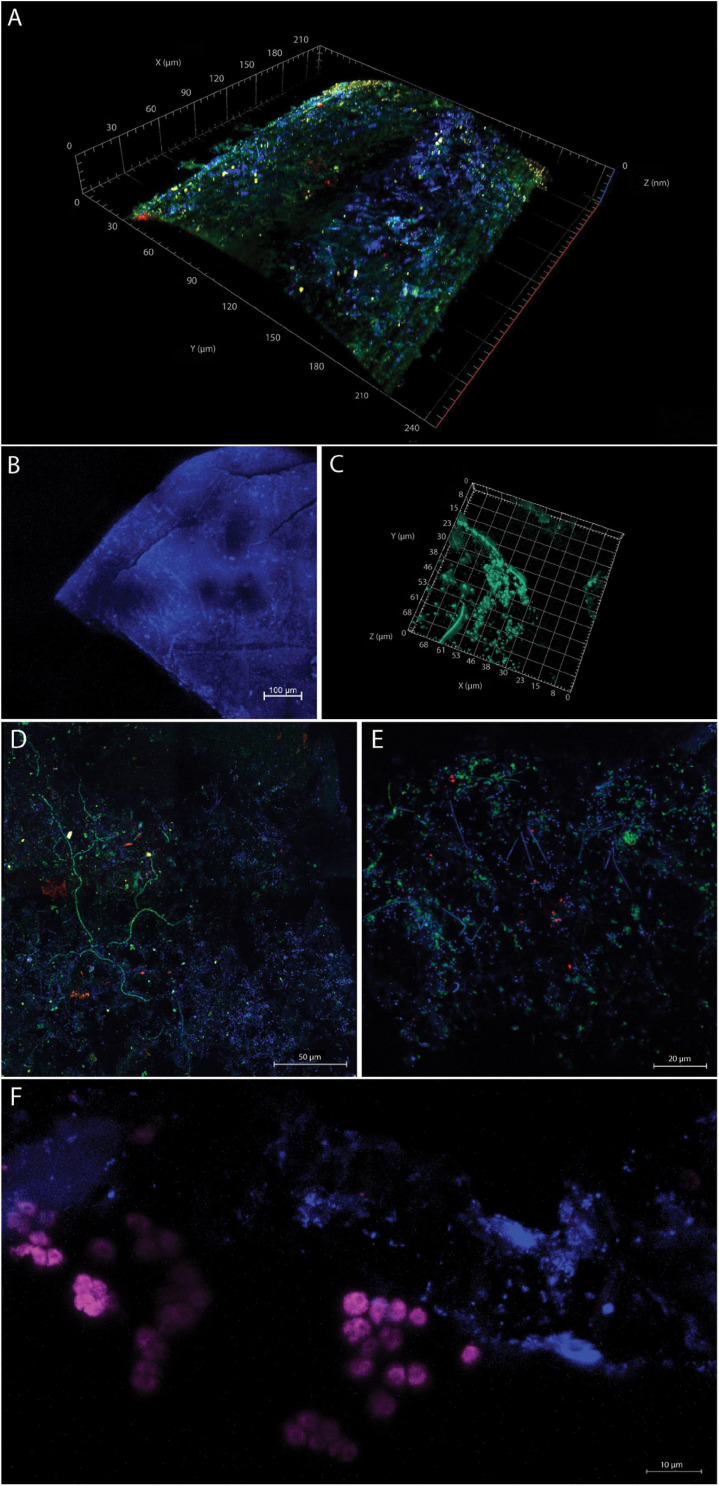
**(A)** Confocal laser scanning **(A,B,D–F)** and superresolution-structured illumination micrographs **(C)** of microbial communities on MPD. The images **(A,C–F)** were scanned as z-stack with a 63X/1.4 objective lens. For image **(B)**, a 10X/0.3 objective lens was used. Images **(D–F)** were visualized as maximum-intensity projection with the following stack sizes: **(D)** 12.6 μm, **(E)** 14 μm, and **(F)** 18.5 μm. **(A)** Confocal laser scanning micrograph of a section of a plastic thread visualized as three-dimensional reconstruction. The micrograph shows DAPI-stained cells in blue, Eukarya hybridized with probe EUK516 in red, Archaea hybridized with probe Arch915 in yellow, and Bacteria hybridized with (EUB I) in green on the surface of the plastic particle. **(B)** Confocal laser scanning overview image showing the surface topography of a sheet-like PE microplastic particle as maximum-intensity projection of a 261-μm-thick z-stack, with prokaryotic and eukaryotic cells stained with DAPI (note that single microbes appear as bright white-blue spheres and rods while the plastic emits a relatively strong blue autofluorescence signal). **(C)** Superresolution-structured illumination micrograph of SYBR green-stained cells on the same particle as in **(B)** shown as a three-dimensional reconstruction close up [note that the field of view is not the same as in **(B)**]. **(D)** DAPI-stained cells (blue), Eukarya hybridized with probe EUK516 (red), Archaea hybridized with probe Arch915 (yellow), and Bacteria hybridized with probe EUB338-I (green; the polymer of this plastic piece remained undetermined). **(E)** Verrucomicrobia were identified by probe EUB338-III (shown in red) on a PE plastic piece. Numerous other microbes (probe EUB338-I, green; DAPI, blue) were detected. **(F)** DAPI-stained cells (blue) and Eukarya hybridized with probe EUK516 (shown in purple) on an unidentified MPD.

Prokaryotic and eukaryotic cells were visualized on different plastic particles to assess the structure and density of microbial biofilms, e.g., location of cells on the smooth surface versus cracks as well as to visualize the diversity of microorganisms attached to plastic particles. DAPI staining revealed dense multilayer microbial biofilms on the particles (Figure 2). Cell abundances were highest on even surfaces, and we did not observe a denser biofilm in the cracks of the plastic, but only a few cells. We applied combinations of probes targeting Bacteria (EUB338-I and EUB338-III), Eukarya (EUK516), Archaea (Arch915), and DAPI staining of total cells and visualized the signals by using 3D confocal laser scanning microscopy. On all of the plastic particles, we observed microbial colonization predominantly composed of Bacteria. We observed colonization by Eukarya, and although present, the abundance of archaeal cells appeared minor. The colonization of plastic particles was patchy and contained microcolonies, with specific organisms grouping together, especially apparent for Eukarya (Figure 2F). Exemplary images of the biofilms are shown in Figure 2 and Supplementary Figure 2. The 3D video animation of microbial communities on Figure 2A is presented in the Supplementary Video.

### 16S rRNA Gene Sequence Analysis of the Microbial Community

The analysis of the 16S rRNA gene sequences revealed a majority of sequences belonging to the domain of Bacteria irrespective of the polymer type from which the DNA was extracted (polyethylene: 95.9 ± 2.1%, polypropylene: 94.7 ± 3.1%, polystyrene: 84.2 ± 16.4%). Within the bacterial community, the most abundant phyla were Proteobacteria, Bacteroidetes, Cyanobacteria, Planctomycetes, Actinobacteria, and Acidobacteria (Supplementary Table 3). Proteobacteria accounted for 54.2 ± 11.2% (PE), 37.8 ± 18.1% (PP), and 27.4 ± 8.6% (PS) and Bacteroidetes 27.6 ± 10.2% (PE), 40.7 ± 17.8% (PP), and 41.3 ± 10.7% (PS) of all 16S rRNA gene sequence reads. Among the Proteobacteria, Alphaproteobacteria 28.7 ± 10.5% (PE), 22.7 ± 10.8% (PP), and 13.8 ± 5.3% (PS) and Gammaproteobacteria 20.0 ± 9.1% (PE), 14.1 ± 12.7% (PP), and 12.5 ± 8.9% (PS) were the most abundant classes, followed by Deltaproteobacteria 5.4 ± 6.9% (PE), 1.0 ± 1.0% (PP), and 1.0 ± 0.8% (PS). The class Bacteroiditia followed by Rhodothermia formed ≥96 and ≥0.5%, respectively, of all Bacteroidetes reads on all plastic types. The class Oxyphotobacteria formed ≥97% of Cyanobacteria reads on all plastic types. Planctomycetes showed a higher diversity on the class level with Planctomycetacia being the most abundant class, 2.9 ± 3.1% (PE), 0.8 ± 1.0% (PP), and 1.3 ± 0.8% (PS) on all polymer types. The OM190, Phycisphaerae, BD7-11, Pla3, and Pla4 lineage were detected on at least one polymer type with >0.1% reads assigned. The most dominant order of Bacteria, irrespective of the plastic type, was Flavobacteriales with 17.8 ± 8.2% (PE), 28.0 ± 18.2% (PP), and 25.9 ± 13.8% (PS). The following orders were present on all plastic types but varied in the rank of abundance. On PE, Rhodobacterales was the second most abundant order (10.4 ± 5.6%) followed by Chitinophagales (5.9 ± 3.3%), Caulobacterales (5.3 ± 3.9%), and Oceanospirillales (5.1 ± 8.1%). On PP, Rickettsiales (7.6 ± 7.4%) was the second most abundant order, followed by Alteromonadales (6.8 ± 9.8%), Cytophagales (6.0 ± 4.4%), and Rhodobacterales (6.0 ± 6.4%). On PS, Cytophagales (9.8 ± 6.6%) was the second most abundant order of Bacteria, followed by Rickettsiales (7.2 ± 5.7%), Chitinophagales (4.9 ± 2.5%), and Alteromonadales (4.8 ± 6.0%). Archaeal sequences accounted for 5.9 ± 10.5% of all sequences on PS, in contrast to PE and PP, where archaeal read abundance was 0.2 ± 0.3% and 0.2 ± 0.4%, respectively. The highest percentage of reads assigned to Archaea on PE was 1.3%, on PP 1.4%, and on PS 31.9%. On PS, most of the reads identified as Archaea were assigned to Thaumarchaeota (5.7 ± 8.4%), followed by Nanoarchaeota phylum (0.3 ± 0.6%). Among the remaining sequences, we detected reads classified as Euryarchaeota, Altiarchaeota, Crenarchaeota, and Diapherotrites. Nearly all sequences within the Thaumarchaeota phylum on PS belonged to the class of

**FIGURE 3:**
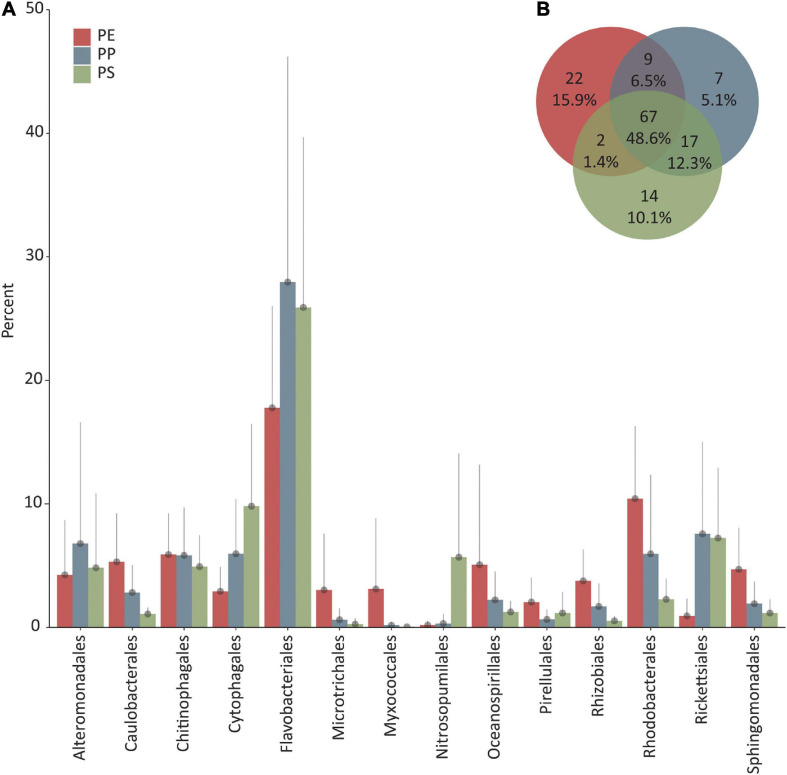
**(A)** Top 10 dominant orders (percentage of total 16S rRNA gene read counts) per plastic type (PE, polyethylene; PP, polypropylene; PS, polystyrene). Error bars depict the standard deviation, and only the upper bar is shown. **(B)** Venn diagram showing the unique and shared number and percentage of the 100 most abundant orders of each plastic type.

Nitrososphaeria, order of Nitrosopumilales. Sequences of the Nanoarchaeota phylum belonged mainly to the Woesearchaeia class. Based on presence/absence of the most abundant orders, the microbial community was similar on all plastic types. However, the number of different taxa detected on the order level was different when also accounting for less abundant orders. When considering the top 100 assigned order levels, 48.6% were shared among all plastic types, 15.9% were unique to PE, 10.1% were unique to PS, and 5.1% to PP (Figure 3).

**FIGURE 4:**
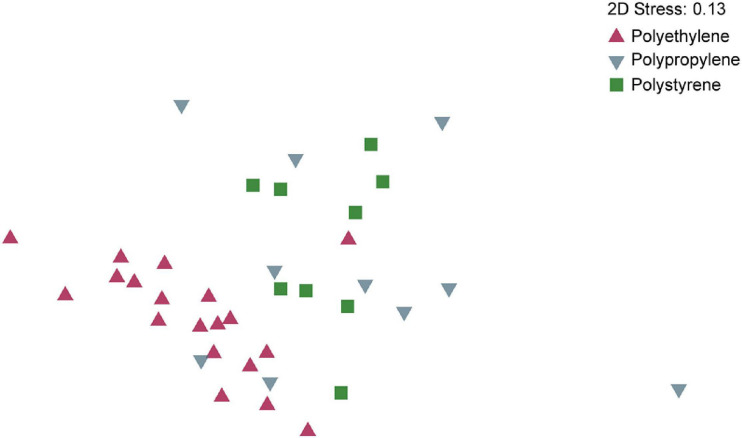
Non-metric multidimensional scaling (NMDS) of microbial communities on OTU level based on 16S rRNA gene sequencing from biofilms colonizing polyethylene, polystyrene, and polypropylene plastic polymers. nMDS on order level yielded similar results (Supplementary Table 1).

We observed significant differences in microbial community composition when comparing the different polymer types (38 identified particles were used). Multivariate statistics (PERMANOVA) revealed that on both, OTU and order level, the microbial composition of PE differed significantly from the microbial community of PP and PS (in all cases *p* < 0.001) while the microbial communities on PP and PS were only significantly different on the OTU level (*p* = 0.025) but not on the order level (*p* = 0.14) (Supplementary Table 1). PERMDISP analysis revealed significant differences in dispersion on order level (*p* = 0.017) but not on OTU level (*p* = 0.059). The significant difference on order level was due to PP samples having a higher dispersion than PE and PS samples. However, combining PP and PS samples to one group and comparing this to PE samples, there was no significant difference in dispersion. Thus, while differences in dispersion between polymer types may contribute to the significant differences in the community composition as shown by PERMANOVA, dispersion had no influence on the detected community composition between PE and the other two polymer types. SIMPER analysis on the order level showed that the main contributors for similarity among all particles classified as PE were Flavobacteriales, Rhodobacterales, and Chitinophagales (ordered by contribution). Among particles classified as PP, these were Flavobacteriales, Chitinophagales, and Rickettsiales, and for PS, these were Flavobacteriales, Cytophagales, and Rickettsiales. The average dissimilarity between PE and PP was 44.2%, with main contributors Rickettsiales, Flavobacteriales, Alteromonadales, and Rhodobacterales. Between PE and PS, the average dissimilarity was 42.6% with Rickettsiales, Nitrosopumilales, Flavobacteriales, and Cytophagales being the main contributors to the observed difference. Between PP and PS, the average dissimilarity was 39.8%, with Nitrosopumilales, Alteromonadales, Rickettsiales, and Flavobacteriales as main contributors to dissimilarity. Bray-Curtis similarity indices were calculated for all samples and visualized by non-metric multidimensional scaling (nMDS) (Figure 4), showing a clear separation of communities on PE and PS, and an overlap of PP and PS samples. PP samples showed higher dispersion than the other polymer types, in line with the results from the dispersion test with PERMDISP.

### Hydrocarbon-Degrading Bacteria

**FIGURE 5:**
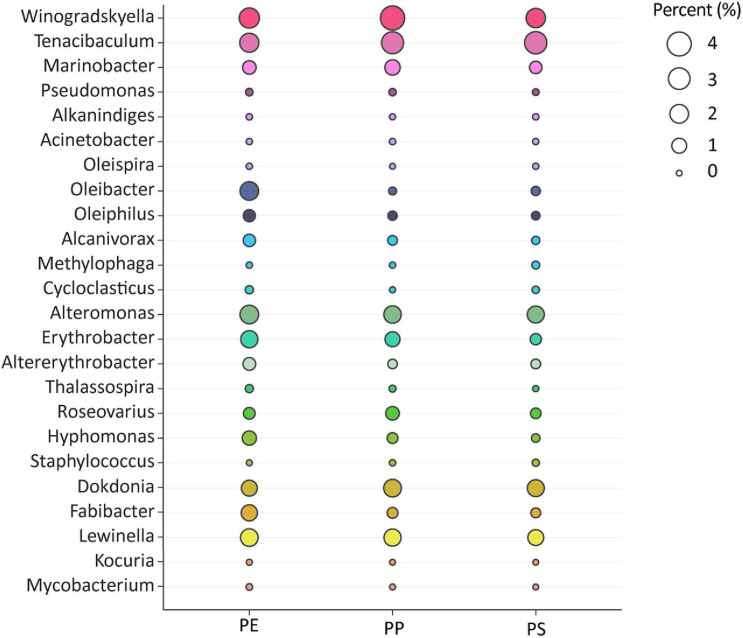
Bacterial genera with members involved in hydrocarbon or oil degradation. Their abundance is presented per plastic type. Sizes of the circles represent rounded percentage of 16S rRNA gene sequence reads assigned to the different taxa.

Among the microbial community, we identified 24 genera previously described to include hydrocarbon-degrading bacteria (HCB). In total, these accounted for up to 17.4% (PE), 16.3% (PP), and 13.0% (PS) of all 16S rRNA gene sequences (Figure 5). All 24 genera were present on all plastic types except for the genus *Kocuria*, which was not detected on PS. The *Winogradskyella* genus accounted for 2.5 ± 1.7% (PE), 4.1 ± 5.5% (PP), and 2.3 ± 1.5% (PS) and the *Tenacibaculum* genus accounted for 2.1 ± 2.7% (PE), 3.2 ± 4.2% (PP), and 3.3 ± 2.7% (PS) of all the 16S rRNA gene reads. *Winogradskyella* and *Tenacibaculum*, both belonging to the *Flavobacteriales* order, were the most abundant genera retrieved from all plastic types. Other HCB genera, accounting for 1.5% of total sequences on at least one of the polymer types were *Alteromonas* 2.0 ± 2.6% (PE), 1.6 ± 2.7% (PP), and 1.6 ± 1.5% (PS); *Oleibacter* 2.1 ± 7.8% (PE), 0.1 ± 0.1% (PP), and 0.2 ± 0.1% (PS); *Lewinella* 1.7 ± 1.5% (PE), 1.6 ± 1.1% (PP), and 1.3 ± 0.6% (PS); *Erythrobacter* 1.7 ± 1.5% (PE), 1.0 ± 1.2% (PP), and 0.4 ± 0.4% (PS); and *Dokdonia* 1.2 ± 1.4% (PE), 1.7 ± 1.7% (PP), and 1.5 ± 1.3% (PS). The HCB genera *Fabibacter*, *Marinobacter*, *Roseovarius*, *Altererythrobacter*, *Alcanivorax*, *Hyphomonas*, and *Oleiphilus* were detected at least on one polymer type with a relative abundance of more than 0.5% of all reads.

## Discussion

### Distribution of Polymers

Semienclosed basins such as the Mediterranean Sea are hotspot areas where MPD accumulates at the sea surface but also in sediments (Vianello et al., 2013; Cozar et al., 2015; Suaria et al., 2016). We sampled floating microplastic in a bay of the Mediterranean island of Elba and found that almost ½ of the polymer fragments were PE and about ¼ were PP and PS. Our sampling technique was different when compared with commonly applied protocols for sampling floating MPD; i.e, we used a handheld net with a mesh size of ∼150 μm, while commonly, neuston/manta nets with a mesh size >300 μm are applied (Prata et al., 2019), and we also did not sample quantitatively (i.e., we did not sample a defined sea surface area). Yet, our results are similar to previous findings from the Mediterranean Sea. In an extensive *trans-*Mediterranean wide study on floating plastic, PE was the most abundantly detected polymer type (∼50% of all-polymer particles), followed by PP (∼16%) (Suaria et al., 2016). Although less PS was on average detected (∼3%), it was found to account for up to 30% in single samplings. This MPD distribution pattern has been found globally and is linked to the production figures of plastics (Geyer et al., 2017; Erni-Cassola et al., 2019b; Wayman and Niemann, 2021). We thus argue that the similarities in polymer ID indicate that our findings are not extraordinary but generally applicable for floating MPD particles in the Mediterranean Sea and possibly other ocean areas, too.

### Biofilms on Marine Plastic Surfaces

Plastic particles in the marine environment provide an unusual surface for microbial attachment and a surface to grow on. Floating plastic particles are commonly colonized by an abundance of microbes (Basili et al., 2020). In this study, we investigated the microbial composition of biofilms on plastic particles which have been floating in the Mediterranean Sea for an unknown period of time, though their state of fragmentation suggests that they have not been littered recently. In the following, we term such types of plastics “wild plastic” (in contrast to intentionally released/installed plastics, e.g., to monitor colonization of microbes on plastic). Several studies investigated the formation of biofilms on installed polymers, for example on weathered and non-weathered PE exposed to Mediterranean seawater (Erni-Cassola et al., 2019a), on PE exposed to different water depths in the Yellow Sea (Tu et al., 2020), on PET bottles installed in the North Sea (Oberbeckmann et al., 2016) and on PE exposed to coastal sediments (Harrison et al., 2014). Though knowledge on the succession of microbial communities on plastic surfaces is undoubtedly necessary, “wild plastics” feature biofilm climax states and allow investigating potential long-term microbe-plastic interactions. However, investigations of microbial communities on single wild plastic debris items as done in this study are less frequent (Zettler et al., 2013; Oberbeckmann et al., 2014; De Tender et al., 2015; Basili et al., 2020). Our CLSM analysis demonstrates that all investigated plastic particles featured a dense biofilm on the surface. We found members of all three domains of life (Eukarya, Bacteria, and Archaea) of which Bacteria were seemingly the most abundant biofilm members (Figure 2). By applying several different FISH probes, we detected different taxa of Bacteria, indicating the applicability of this method to visualize life on single plastic particles, similar to the characterization of microbes on other minuscule three-dimensional substrates such as sand grains (Probandt et al., 2018). For visualization of biofilms and plastic surfaces, several studies have applied scanning electron microscopy (SEM) (Carson et al., 2013; Zettler et al., 2013; Bryant et al., 2016; Kirstein et al., 2018, 2019; Miralles et al., 2018). With SEM analysis, Eukarya were identified, of which diatoms are early and/or dominant colonizers (Eich et al., 2015; Dudek et al., 2020; Zhao et al., 2020). However, SEM does not allow the identification of the attached prokaryotes. CLSM analysis, in combination with FISH, is a much more powerful technique, as it allows labeling of different prokaryotic and eukaryotic taxa with specific probes and visualization of these on a surface (Schlundt et al., 2020). Application of FISH and CLSM allows identification of the structure of the biofilms, localization of specific microbial taxa, microcolonies, and identification of microniches. Furthermore, physical interactions of identified populations can be studied. We observed numerous cracks and fissures on almost all plastic particles, but we did not observe that cracks harbor more microbes in comparison with smooth surfaces. This observation is counterintuitive, as cracks provide sheltered spaces that could shield microbes from, e.g., abrasion (Zettler et al., 2013). On sand grains, for example, microbial communities were observed to be patchy, but protected niches were more densely populated, while fewer microbes were observed on exposed surfaces (Probandt et al., 2018; Ahmerkamp et al., 2020). However, comparing micrographs of microbial communities on sand grains (Probandt et al., 2018) with our analysis, it appears that many more cells were present in the multilayer biofilm on plastic particles. Our findings of higher cell abundances on smooth surfaces and not in cracks, might result from recent and ongoing fragmentation of the plastic pieces (caused by shear stress induced by wave action and weathering induced by UV radiation). Along this line, it seems likely that the cracks are a rather recently created niche for colonization and that cracks open up further until a fragment is fully separated. Additionally, the shape of the MPD fractures is different in comparison with sand grains, which are spherical. This might support ventilation of the protected areas on sand grains providing microbes in these niches with necessary oxygen (Ahmerkamp et al., 2020). However, further research and visualization of communities on the plastic need to confirm this.

### Polymer Types Selecting for Distinct Microbial Communities

On the taxonomic level of order and OTU, our results demonstrate that specific plastic types support biofilms with distinct microbial communities, though a large number of taxonomic groups may be shared between different polymers. For PE, PP, and PS analyzed here, these distinctions are caused by variations in relative abundance (frequency of sequences) and to a lesser degree by presence/absence of specific groups. Most importantly, our results show that PE supports a significantly different microbial community than PP and PS on both order and OTU level, while the difference between PP and PS was only significant on the OTU level. We observed higher dispersion in PP samples, which might influence the PERMANOVA test results for differences in community composition. However, nMDS confirmed that there is indeed a difference in community composition (i.e., location) between PE and the remaining polymer types on both OTU and order level. Concerning the significant differences on order level between PP and PS, these were likely influenced by the different dispersions in these groups. Long-term flow-through incubations of several polymers and glass revealed that communities on plastics are different from control surface and communities differ between some polymers (Kirstein et al., 2018). Furthermore, a similar result as shown here was found in a study investigating PS, PE, and PP in the coastal Atlantic, where PS featured bacterial assemblages different to those on PE and PP (Frère et al., 2018). However, just as we observed, the overlap of assemblages between PE and PP was considerable. Structurally, PE and PP are polyolefins consisting of long carbon-carbon backbones of repetitive ethylene and propylene subunits, respectively. In contrast, polystyrene comprises aromatic rings. The chemical structure, as well as surface properties of these polymers differ and as a consequence, different daughter products are generated through UV radiation (Gewert et al., 2015, 2018). We argue that the differential surface properties of the polymer (virgin structure and/or modified through UV radiation) and/or leaching of daughter products from UV-degradation select for differential microbial communities. Furthermore, these communities might directly or indirectly interact with the plastic substrate including potential biodegradation of the virgin or weathered plastic (Wayman and Niemann, 2021). Also, others have demonstrated the presence of plastic-specific microbial biofilms on different plastic types (De Tender et al., 2017; Oberbeckmann et al., 2018; Woodall et al., 2018) or that MPDs feature a distinct microbial community compared with seawater (Bryant et al., 2016; De Tender et al., 2017; Kettner et al., 2017; Dussud et al., 2018b; Frère et al., 2018; Kirstein et al., 2018) and in particular when only considering the part of the biofilm that is in direct contact and tightly adhered to the polymer (Kirstein et al., 2019). Besides, it has been suggested that microbial communities are more polymer specific during the early stages of colonization (Pinto et al., 2019). Currently, it remains challenging to determine if specific polymers select for a specific microbial community across a broader scale of environmental settings. Variations in microbial communities may originate from investigations conducted in different geographical locations with different environmental conditions (Oberbeckmann et al., 2018; Kesy et al., 2019; Li et al., 2019; Muthukrishnan et al., 2019; Erni-Cassola et al., 2020; Wright et al., 2020). Moreover, the differences in experimental design make it challenging to clearly determine in how far differences in microbial community structure depend on the polymer type.

### Microbial Community Composition—Bacteria

Several of the taxonomic orders and families detected here have been reported as preferential or early colonizers on different submerged surfaces, including plastics. In our samples, 67 of the top 100 orders forming the core community, were shared between PE, PP, and PS. These taxa have commonly been found on floating MPD and have thus been referred to as a general plastic biofilm community (Kirstein et al., 2019). *Flavobacteriaceae* and *Rhodobacteraceae* were found as core members of the biofilms on polyethylene sheets and dolly ropes exposed to the seafloor in a harbor and offshore of the North Sea (De Tender et al., 2017), on PET bottles in the North Sea surface waters (Oberbeckmann et al., 2016), on plastic surfaces of PE and PP in the North Atlantic Ocean (Zettler et al., 2013), as well as on MPD sampled from the North Pacific Gyre (Bryant et al., 2016). In addition, some of the shared taxa are general colonizers of hard surfaces. For example, members of the order *Rhodobacterales*, commonly found on plastics as early colonizers (Dang and Lovell, 2000; Dang et al., 2008), have also been found to remain core microbes of more mature biofilms on plastic (Tu et al., 2020). Furthermore, *Cyclobacteriaceae* have shown a preferential attachment to plastic (PE and PP) over natural surfaces, yet this was found in a freshwater environment (Miao et al., 2019). Similar to our findings, *Rhodobacteraceae*, *Flavobacteriaceae*, and *Alteromonadaceae* were among the most abundant families found on PP (and PVC) in coastal waters of the Yellow sea and South-China sea after a year of exposure (Xu et al., 2019). Although *Saprospiraceae* have been detected on various plastic types (Oberbeckmann et al., 2018), this clade may be more specific to PS (Kirstein et al., 2019). However, we observed *Saprospiraceae* on all polymer types as one of the most abundant taxa. In contrast to previous studies where members of the family *Vibrionaceae* were typically found in biofilms on plastics (Zettler et al., 2013; Kirstein et al., 2016; Frère et al., 2018), we did not observe high abundance of *Vibrio* species. *Vibrio spp*. seem to prefer a free-living lifestyle rather than being particle-associated (Liang et al., 2019; Li et al., 2020). However, *Vibrio spp.* have been reported to degrade a composite polymer of polyvinyl alcohol-low linear density PE in exposure experiments with this polymer as the sole carbon source (Raghul et al., 2014). *Vibrio* spp. was also shown to degrade plastic bottle waste, when this was the only available carbon source (Sarkhel et al., 2019).

### Abundance and Distribution of Hydrocarbon Degraders

Plastics comprise hydrocarbon-like compounds with simple (polyolefins such as PE and PP) or more complex C–C skeletons (PS featuring aromatic rings). Hydrocarbon-degrading Bacteria (HCB) are hence potential candidates to utilize or cometabolize these types of plastics (Dussud et al., 2018b; Basili et al., 2020; Erni-Cassola et al., 2020). HCB use linear, branched, or aromatic hydrocarbons as sole energy, and carbon source and are often found in oil reservoirs, oil seeps, or oil spills (Joye et al., 2014), contaminated sediments (Kimes et al., 2013) and coal beds (Beckmann et al., 2019), but they are also found ubiquitously in the marine environment (Yakimov et al., 2007; Erni-Cassola et al., 2020; Thompson et al., 2020). Although HCB are often detected on plastic (Wright et al., 2020), previous investigations seldomly focus on the potential role of HCB as MPD degraders (Erni-Cassola et al., 2020). HCB comprise >175 genera (Prince et al., 2010) and feature enzymes such as mono- and dioxygenases or peroxidases (Brzeszcz and Kaszycki, 2018). In principle, these might be able to attack the primary polymer structure of plastic and/or further degrade daughter products generated through weathering of the primary polymers (Wayman and Niemann, 2021). The genomic potential of *Alcanivorax*, for example, reveals the presence of several alkane degradation genes (Brooijmans et al., 2009). Furthermore, the genome of *Alcanivorax* sp. *24*, isolated from marine plastic contains three alk B genes, two FAD-dependent monooxygenases, 13 esterases, and 17 peroxidases, possibly involved in the biodegradation of polymers (Zadjelovic et al., 2020). An isolated strain closely related to *Alcanivorax borkumensis* was found to degrade LDPE (Delacuvellerie et al., 2019). Previous studies have also found HCB on different polymer types (Zettler et al., 2013; Oberbeckmann et al., 2016; Debroas et al., 2017; Dussud et al., 2018b; Pinto et al., 2019; Erni-Cassola et al., 2020). To our knowledge, the highest abundance of HCBs (34% of all total sequences on polymer samples) was found in a study investigating microbial colonization on PE, aged PE, PE with pro-oxidant additives, PHBV, and polyester in a flow-through reactor with Mediterranean seawater (Dussud et al., 2018a). This study also showed that *Alcanivorax* sp., *Alteromonas* sp., *Marinobacter* sp., and *Oleiphilus messinensis* were more abundant on any of the tested PE types compared with seawater and were particularly more abundant during the primary phase of colonization. Furthermore, consortia of marine HCB were shown to efficiently degrade PET in microcosms (Denaro et al., 2020). We identified genera which contain hydrocarbon and oil degraders on all polymer types, with a minimum of 13% of total 16S rRNA gene reads. However, the genera analyzed here include HCBs but not necessarily all detected sequences of these genera are HCBs. Higher abundance of HCBs have been found in the early stages of biofilm formation (Dussud et al., 2018b; Erni-Cassola et al., 2020). Nonetheless, in our samples with most likely fully mature biofilms, we identified 24 genera of hydrocarbon degraders (dominantly belonging to the *Flavobacteriales* order), some of which were very abundant with ≥4% of all 16S rRNA gene reads. HCBs with the highest relative abundance in our study were *Winogradskyella* and *Tenacibaculum*, which showed similar relative read abundances on all plastic types. *Winogradskyella* has been suggested as a specific group in biofilms on sedimentary plastic (Delacuvellerie et al., 2019), although it was detected in low numbers. *Winogradskyella* has also been found on plastic exposed in the North Sea (Kirstein et al., 2019). *Tenacibaculum* was previously found as the most abundant genus in biofilms on PET bottles in the North Sea (Oberbeckmann et al., 2016). The genus *Tenacibaculum* has also been detected on different polymer types in various abundances in the North Pacific (Bryant et al., 2016), in the North Sea (De Tender et al., 2017), North Atlantic (Zettler et al., 2013), as well as in the Mediterranean Sea (Dussud et al., 2018a). Some of the genera detected here, i.e., *Oleispira*, *Oleiphilus*, *Alcanivorax*, *Marinobacter*, and *Cycloclasticus*, are even obligate hydrocarbon degraders (Brooijmans et al., 2009), and as in the case of *Alcanivorax* were found to be involved in plastic degradation (see above). Finally, *Erythrobacteraceae* and *Hyphomonadaceae* both of which were previously also found on PE and PS (Oberbeckmann et al., 2018), were defined as members of the core community on plastics (De Tender et al., 2017).

### Microbial Community Composition—Archaea

Most studies on plastic colonization and biofilm formation focused mainly on Bacteria and/or Eukarya, while Archaea, when targeted, were encountered at lower abundances (Oberbeckmann et al., 2016; Dussud et al., 2018a; Woodall et al., 2018); i.e., overall, they have received less attention as plastic colonizers. In our FISH analyses, we also observed relatively few archaeal cells colonizing plastic particles. This was supported by sequencing, where we detected fewer archaeal reads in comparison with bacteria. We detected a higher archaeal relative read abundance on PS compared with PP and PE. The detected Archaea mostly belonged to the phylum Thaumarchaeota (family *Nitrosopumilaceae*). Their preference for one polymer type (PS) above the other types remains unclear. In the marine water column, *Nitrosopumilaceae* mediate aerobic ammonia oxidation and CO_2_ fixation (Qin et al., 2017), and hence might contribute to the overall nitrogen cycling in the biofilm. In contrast to our findings, previous research has found an abundance of Crenarchaeota as the sole archaeal phylum on plastic macrodebris in the deep sea (Woodall et al., 2018). On PET bottles in the North Sea, Marine group II of the phylum Euryarchaeota was detected among the top 25 families (Oberbeckmann et al., 2016). Typically, archaea of Marine group II are abundant in seawater (Pernthaler et al., 2002b; Galand et al., 2010), but so far, their environmental functioning remains elusive (Zhang et al., 2015). Archaea were also reported in biofilms attached to MPD from the North Atlantic (Debroas et al., 2017). Nevertheless, though archaeal reads were highly abundant, no significant association to any of the investigated polymer types could be found. Comparing the previous findings of Archaea on plastic and considering our results, it appears that there is no archaeal analogy to the bacterial core community associated with plastics. It thus remains speculative if our findings of higher abundances of Archaea belonging to the *Nitrosopumilus* clade on PS is specific for this polymer type. However, we suggest that future studies should also target Archaea to further investigate their role in MPD-associated biofilms.

## Conclusion

Our results of 16S rRNA amplicon gene sequencing provide evidence that diverse microbial communities thrive on different types of MPD. However, as shown by FISH analysis, these microbial biofilms are not homogenous and contain microcolonies and clusters of specific taxa. Our sequencing analysis provides a strong indication that different polymer types may select for differential communities. Our results furthermore show that less-abundant members of the microbial community (that are probably not part of the early colonizers) and differences in relative abundances of microbial taxa seemingly determine the specificity of a microbial community on a given polymer type. Our results also suggest that the most-abundant members of the MPD-associated biofilms do not play an important role in potential plastic degradation. Instead, our findings of the high abundance of genera encompassing HCB suggest that these groups could play a potential role in plastic degradation as these microbes have the necessary set of enzymes to degrade complex polymers. However, the functioning of these communities in MPD-associated biofilms remains to be elucidated in future studies. In order to study plastic degradation and the potential role of specific microorganisms in plastic degradation, we suggest that future studies should focus on (i) metagenomics and metatranscriptomics of microbial communities with MPD (as the sole carbon source) to identify species and genes potentially involved in plastic degradation, and (ii) isolating specific microorganisms for testing their ability to degrade plastics. The latter would also allow to further investigate their biochemical functioning, genetic, and enzymatic potential.

## Data Availability Statement

The sequences have been submitted to the Sequence Read Archive (SRA), bioproject PRJNA698954.

## Author Contributions

HN and AV designed the study and experiments. HN and MG carried out sampling. CL and MW supported sampling efforts. AE, KK, and AV carried out FISH analysis. AE performed confocal laser scanning and superresolution-structured illumination microscopy and analysis. AV, IV, and FM carried out Raman spectroscopy. HJW conducted the statistical analysis. AAA performed bioinformatic analysis. JCE supervised bioinformatic and statistical analyses. AV wrote the manuscript with contribution from all coauthors. HN supervised the project. All authors contributed to the article and approved the submitted version.

## Conflict of Interest

The authors declare that the research was conducted in the absence of any commercial or financial relationships that could be construed as a potential conflict of interest.

## Footnotes

1 https://www.bioinformatics.babraham.ac.uk/projects/fastqc/

2 https://github.com/frederic-mahe/stampa

## Supplementary Material

Supplementary Data Sheet 1 Report of the amplicon sequencing data analysis with Cascabel.

Supplementary Data Sheet 2 Raman Spectra.

Supplementary Video The 3D video animation of microbial communities on marine plastic debris.

Supplementary Figure 1 Marine plastic debris.

Supplementary Figure 2 Direct visualisation of Planctomycetes and other bacteria on plastic particles using CARD-FISH and confocal laser scanning microscopy. **(A)** Planctomycetes were identified by probe Pla46 (shown in red). Numerous other microbes (probe EUB338-I, green; DAPI, blue) were detected. **(B)** DAPI stained cells (shown in blue), and Bacteria identified by probe EUB338-I (in green), Gammaproteobacteria identified by probe GAM42a (shown in red) and Planctomycetes were identified by probe Pla46 (shown in yellow). **(C)** Stained same as **B** on alternative plastic piece. All images where scanned as *z*-stack using a 63×/1.4 objective lens (stack size **A** 12.6 μm, **B** 14 μm, and **C** 18.5 μm) and visualised as maximum intensity projection.

Supplementary Table 1 Statistical analysis.

Supplementary Table 2 FISH probes used in this study.

Supplementary Table 3 Top 10 dominant phyla in total 16S rRNA gene read counts per polymer type.

## References

[B1] Abdala Asbun A. Besseling M. A. Balzano S. Van Bleijswijk J. D. L. Witte H. J. Villanueva L. (2020). Cascabel: a scalable and versatile amplicon sequence data analysis pipeline delivering reproducible and documented results. *Front. Genet.* 11:1329. 10.3389/fgene.2020.489357 PMC7718033 33329686

[B2] Ahmerkamp S. Marchant H. K. Peng C. Probandt D. Littmann S. Kuypers M. M. M. (2020). The effect of sediment grain properties and porewater flow on microbial abundance and respiration in permeable sediments. *Sci. Rep.* 10;3573. 32107429 10.1038/s41598-020-60557-7 PMC7046789

[B3] Asensio-Montesinos F. Anfuso G. Williams A. T. (2019). Beach litter distribution along the western Mediterranean coast of Spain. *Mar. Pollut. Bull.* 141 119–126. 10.1016/j.marpolbul.2019.02.031 30955716

[B4] Basili M. Quero G. M. Giovannelli D. Manini E. Vignaroli C. Avio C. G. (2020). Major role of surrounding environment in shaping biofilm community composition on marine plastic debris. *Front. Mar. Sci.* 7:262. 10.3389/fmars.2020.00262

[B5] Beckmann S. Luk A. W. S. Gutierrez-Zamora M.-L. Chong N. H. H. Thomas T. Lee M. (2019). Long-term succession in a coal seam microbiome during in situ biostimulation of coalbed-methane generation. *ISME J.* 13 632–650. 10.1038/s41396-018-0296-5 30323265 PMC6461797

[B6] Brooijmans R. J. Pastink M. I. Siezen R. J. (2009). Hydrocarbon-degrading bacteria: the oil-spill clean-up crew. *Microb. Biotechnol.* 2 587–594. 10.1111/j.1751-7915.2009.00151.x 21255292 PMC3815313

[B7] Bryant J. A. Clemente T. M. Viviani D. A. Fong A. A. Thomas K. A. Kemp P. (2016). Diversity and activity of communities inhabiting plastic debris in the north pacific gyre. *mSystems* 1:e00024–16. 27822538 10.1128/mSystems.00024-16 PMC5069773

[B8] Brzeszcz J. Kaszycki P. (2018). Aerobic bacteria degrading both n-alkanes and aromatic hydrocarbons: an undervalued strategy for metabolic diversity and flexibility. *Biodegradation* 29 359–407. 10.1007/s10532-018-9837-x 29948519

[B9] Caporaso J. G. Kuczynski J. Stombaugh J. Bittinger K. Bushman F. D. Costello E. K. (2010). QIIME allows analysis of high-throughput community sequencing data. *Nat. Methods* 7 335–336. 20383131 10.1038/nmeth.f.303 PMC3156573

[B10] Carson H. S. Nerheim M. S. Carroll K. A. Eriksen M. (2013). The plastic-associated microorganisms of the North Pacific Gyre. *Mar. Pollut. Bull.* 75 126–132. 10.1016/j.marpolbul.2013.07.054 23993070

[B11] Clunies-Ross P. Smith G. Gordon K. Gaw S. (2016). Synthetic shorelines in New Zealand? Quantification and characterisation of microplastic pollution on Canterbury’s coastlines. *N. Z. J. Mar. Freshw. Res.* 50 1–9.

[B12] Cozar A. Echevarria F. Gonzalez-Gordillo J. I. Irigoien X. Ubeda B. Hernandez-Leon S. (2014). Plastic debris in the open ocean. *Proc. Natl. Acad. Sci. U. S. A.* 111 10239–10244. 24982135 10.1073/pnas.1314705111 PMC4104848

[B13] Cozar A. Sanz-Martin M. Marti E. Gonzalez-Gordillo J. I. Ubeda B. Galvez J. A. (2015). Plastic accumulation in the Mediterranean sea. *PLoS One* 10:e0121762. 10.1371/journal.pone.0121762 25831129 PMC4382178

[B14] Crawford C. B. Quinn B. (2017). “10 – Microplastic identification techniques,” in *Microplastic Pollutants*, eds Crawford C. B. Quinn B. (Amsterdam: Elsevier), 219–267. 10.1016/b978-0-12-809406-8.00010-4

[B15] Dang H. Li T. Chen M. Huang G. (2008). Cross-ocean distribution of Rhodobacterales bacteria as primary surface colonizers in temperate coastal marine waters. *Appl. Environ. Microbiol.* 74 52–60. 10.1128/aem.01400-07 17965206 PMC2223210

[B16] Dang H. Lovell C. R. (2000). Bacterial primary colonization and early succession on surfaces in marine waters as determined by amplified rRNA gene restriction analysis and sequence analysis of 16S rRNA Genes. *Appl. Environ. Microbiol.* 66:467. 10.1128/aem.66.2.467-475.2000 10653705 PMC91850

[B17] de Carvalho C. C. C. R. (2018). Marine biofilms: a successful microbial strategy with economic implications. *Front. Mar. Sci.* 5:126. 10.3389/fmars.2018.00126

[B18] de Haan W. P. Sanchez-Vidal A. Canals M. (2019). Floating microplastics and aggregate formation in the Western Mediterranean Sea. *Mar. Pollut. Bull.* 140 523–535. 10.1016/j.marpolbul.2019.01.053 30803674

[B19] De Tender C. Devriese L. I. Haegeman A. Maes S. Vangeyte J. Cattrijsse A. (2017). Temporal dynamics of bacterial and fungal colonization on plastic debris in the North Sea. *Environ. Sci. Technol.* 51 7350–7360. 10.1021/acs.est.7b00697 28562015

[B20] De Tender C. A. Devriese L. I. Haegeman A. Maes S. Ruttink T. Dawyndt P. (2015). Bacterial community profiling of plastic litter in the belgian part of the North Sea. *Environ. Sci. Technol.* 49 9629–9638. 10.1021/acs.est.5b01093 26204244

[B21] Debroas D. Mone A. Ter Halle A. (2017). Plastics in the North Atlantic garbage patch: a boat-microbe for hitchhikers and plastic degraders. *Sci. Total Environ.* 599-600 1222–1232. 10.1016/j.scitotenv.2017.05.059 28514840

[B22] Delacuvellerie A. Cyriaque V. Gobert S. Benali S. Wattiez R. (2019). The plastisphere in marine ecosystem hosts potential specific microbial degraders including *Alcanivorax borkumensis* as a key player for the low-density polyethylene degradation. *J. Hazard. Mater.* 380:120899. 10.1016/j.jhazmat.2019.120899 31326835

[B23] Denaro R. Aulenta F. Crisafi F. Di Pippo F. Cruz Viggi C. Matturro B. (2020). Marine hydrocarbon-degrading bacteria breakdown poly(ethylene terephthalate) (PET). *Sci. Total Environ.* 749:141608. 10.1016/j.scitotenv.2020.141608 32836129

[B24] Dudek K. L. Cruz B. N. Polidoro B. Neuer S. (2020). Microbial colonization of microplastics in the Caribbean Sea. *Limnol. Oceanogr. Lett.* 5 5–17. 10.1002/lol2.10141

[B25] Dussud C. Hudec C. George M. Fabre P. Higgs P. Bruzaud S. (2018a). Colonization of non-biodegradable and biodegradable plastics by marine microorganisms. *Front. Microbiol.* 9:1571. 10.3389/fmicb.2018.01571 30072962 PMC6058052

[B26] Dussud C. Meistertzheim A. L. Conan P. Pujo-Pay M. George M. Fabre P. (2018b). Evidence of niche partitioning among bacteria living on plastics, organic particles and surrounding seawaters. *Environ. Pollut.* 236 807–816. 10.1016/j.envpol.2017.12.027 29459335

[B27] Edgar R. C. (2010). Search and clustering orders of magnitude faster than BLAST. *Bioinformatics* 26 2460–2461. 10.1093/bioinformatics/btq461 20709691

[B28] Eich A. Mildenberger T. Laforsch C. Weber M. (2015). Biofilm and diatom succession on polyethylene (PE) and biodegradable plastic bags in two marine habitats: early signs of degradation in the pelagic and benthic zone? *PLoS One* 10:e0137201. 10.1371/journal.pone.0137201 26394047 PMC4578875

[B29] Erni-Cassola G. Wright R. J. Gibson M. I. Christie-Oleza J. A. (2019a). Early colonization of weathered polyethylene by distinct bacteria in marine coastal seawater. *Microb. Ecol.* 79 517–526. 10.1007/s00248-019-01424-5 31463664 PMC7176602

[B30] Erni-Cassola G. Wright R. J. Gibson M. I. Christie-Oleza J. A. (2020). Early colonization of weathered polyethylene by distinct bacteria in marine coastal seawater. *Microb. Ecol.* 79 517–526. 10.1007/s00248-019-01424-5 31463664 PMC7176602

[B31] Erni-Cassola G. Zadjelovic V. Gibson M. I. Christie-Oleza J. A. (2019b). Distribution of plastic polymer types in the marine environment; a meta-analysis. *J. Hazard. Mater.* 369 691–698. 10.1016/j.jhazmat.2019.02.067 30826562

[B32] Filgueiras A. V. Gago J. Campillo J. A. León V. M. (2019). Microplastic distribution in surface sediments along the Spanish Mediterranean continental shelf. *Environ. Sci. Pollut. Res.* 26 21264–21273. 10.1007/s11356-019-05341-5 31119537

[B33] Frère L. Maignien L. Chalopin M. Huvet A. Rinnert E. Morrison H. (2018). Microplastic bacterial communities in the bay of brest: influence of polymer type and size. *Environ. Pollut.* 242 614–625. 10.1016/j.envpol.2018.07.023 30014939

[B34] Frère L. Paul-Pont I. Moreau J. Soudant P. Lambert C. Huvet A. (2016). A semi-automated Raman micro-spectroscopy method for morphological and chemical characterizations of microplastic litter. *Mar. Pollut. Bull.* 113 461–468. 10.1016/j.marpolbul.2016.10.051 27837909

[B35] Galand P. E. Gutiérrez-Provecho C. Massana R. Gasol J. M. Casamayor E. O. (2010). Inter-annual recurrence of archaeal assemblages in the coastal NW Mediterranean Sea (blanes bay microbial observatory). *Limnol. Oceanogr.* 55 2117–2125. 10.4319/lo.2010.55.5.2117

[B36] Gewert B. Plassmann M. Sandblom O. Macleod M. (2018). Identification of chain scission products released to water by plastic exposed to ultraviolet light. *Environ. Sci. Technol. Lett.* 5:272–276. 10.1021/acs.estlett.8b00119

[B37] Gewert B. Plassmann M. M. Macleod M. (2015). Pathways for degradation of plastic polymers floating in the marine environment. *Environ. Sci.* 17 1513–1521. 10.1039/c5em00207a 26216708

[B38] Geyer R. Jambeck J. R. Law K. L. (2017). Production, use, and fate of all plastics ever made. *Sci. Adv.* 3:e1700782. 10.1126/sciadv.1700782 28776036 PMC5517107

[B39] Giani D. Baini M. Galli M. Casini S. Fossi M. C. (2019). Microplastics occurrence in edible fish species (*Mullus barbatus* and *Merluccius merluccius*) collected in three different geographical sub-areas of the Mediterranean Sea. *Mar. Pollut. Bull.* 140 129–137. 10.1016/j.marpolbul.2019.01.005 30803626

[B40] Gilan I. Hadar Y. Sivan A. (2004). Colonization, biofilm formation and biodegradation of polyethylene by a strain of Rhodococcus ruber. *Appl. Microbiol. Biotechnol.* 65 97–104. 15221232 10.1007/s00253-004-1584-8

[B41] Gonçalves C. Martins M. Sobral P. Costa P. M. Costa M. H. (2019). An assessment of the ability to ingest and excrete microplastics by filter-feeders: a case study with the *Mediterranean mussel*. *Environ. Pollut.* 245 600–606. 10.1016/j.envpol.2018.11.038 30476889

[B42] Gravouil K. Ferru-Clément R. Colas S. Helye R. Kadri L. Bourdeau L. (2017). Transcriptomics and lipidomics of the environmental strain rhodococcus ruber point out consumption pathways and potential metabolic bottlenecks for polyethylene degradation. *Environ. Sci. Technol.* 51 5172–5181. 10.1021/acs.est.7b00846 28345896

[B43] Harrison J. P. Schratzberger M. Sapp M. Osborn A. M. (2014). Rapid bacterial colonization of low-density polyethylene microplastics in coastal sediment microcosms. *BMC Microbiol.* 14:232. 10.1186/s12866-014-0232-4 25245856 PMC4177575

[B44] Herrero Acero E. Ribitsch D. Steinkellner G. Gruber K. Greimel K. Eiteljoerg I. (2011). Enzymatic surface hydrolysis of PET: effect of structural diversity on kinetic properties of cutinases from thermobifida. *Macromolecules* 44 4632–4640. 10.1021/ma200949p

[B45] Jacquin J. Cheng J. Odobel C. Pandin C. Conan P. Pujo-Pay M. (2019). Microbial ecotoxicology of marine plastic debris: a review on colonization and biodegradation by the “Plastisphere”. *Front. Microbiol.* 10:865. 10.3389/fmicb.2019.00865 31073297 PMC6497127

[B46] Jambeck J. R. Geyer R. Wilcox C. Siegler T. R. Perryman M. Andrady A. (2015). Plastic waste inputs from land into the ocean. *Science* 347 768–771. 10.1126/science.1260352 25678662

[B47] Joye S. B. Teske A. P. Kostka J. E. (2014). Microbial dynamics following the macondo oil well blowout across gulf of mexico environments. *BioScience* 64 766–777. 10.1093/biosci/biu121

[B48] Kaiser D. Kowalski N. Waniek J. J. (2017). Effects of biofouling on the sinking behavior of microplastics. *Environ. Res. Lett.* 12:124003. 10.1088/1748-9326/aa8e8b

[B49] Kane I. A. Clare M. A. Miramontes E. Wogelius R. Rothwell J. J. Garreau P. (2020). Seafloor microplastic hotspots controlled by deep-sea circulation. *Science* 368:eaba5899. 10.1126/science.aba5899 32354839

[B50] Kesy K. Oberbeckmann S. Kreikemeyer B. Labrenz M. (2019). Spatial environmental heterogeneity determines young biofilm assemblages on microplastics in baltic sea mesocosms. *Front. Microbiol.* 10:1665. 10.3389/fmicb.2019.01665 31447791 PMC6696623

[B51] Kettner M. T. Rojas-Jimenez K. Oberbeckmann S. Labrenz M. Grossart H.-P. (2017). Microplastics alter composition of fungal communities in aquatic ecosystems. *Environ. Microbiol.* 19 4447–4459. 10.1111/1462-2920.13891 28805294

[B52] Kimes N. E. Callaghan A. V. Aktas D. F. Smith W. L. Sunner J. Golding B. (2013). Metagenomic analysis and metabolite profiling of deep-sea sediments from the Gulf of Mexico following the Deepwater Horizon oil spill. *Front. Microbiol.* 4:50. 10.3389/fmicb.2013.00050 23508965 PMC3598227

[B53] Kirstein I. V. Kirmizi S. Wichels A. Garin-Fernandez A. Erler R. Löder M. (2016). Dangerous hitchhikers? Evidence for potentially pathogenic Vibrio spp. on microplastic particles. *Mar. Environ. Res.* 120 1–8. 10.1016/j.marenvres.2016.07.004 27411093

[B54] Kirstein I. V. Wichels A. Gullans E. Krohne G. Gerdts G. (2019). The Plastisphere – Uncovering tightly attached plastic “specific” microorganisms. *PLoS One* 14:e0215859. 10.1371/journal.pone.0215859 31013334 PMC6478340

[B55] Kirstein I. V. Wichels A. Krohne G. Gerdts G. (2018). Mature biofilm communities on synthetic polymers in seawater – Specific or general? *Mar. Environ. Res.* 142 147–154. 10.1016/j.marenvres.2018.09.028 30337052

[B56] Li B. Liu J. Zhou S. Fu L. Yao P. Chen L. (2020). Vertical variation in Vibrio community composition in Sansha Yongle Blue Hole and its ability to degrade macromolecules. *Mar. Life Sci. Technol.* 2 60–72. 10.1007/s42995-019-00003-4

[B57] Li W. Zhang Y. Wu N. Zhao Z. Xu W. A. Ma Y. (2019). Colonization characteristics of bacterial communities on plastic debris influenced by environmental factors and polymer types in the haihe estuary of Bohai Bay, China. *Environ. Sci. Technol.* 53 10763–10773. 10.1021/acs.est.9b03659 31441645

[B58] Liang J. Liu J. Wang X. Lin H. Liu J. Zhou S. (2019). Spatiotemporal dynamics of free-living and particle-associated vibrio. *Appl. Environ. Microbiol.* 85 e00217–e00219. 30824453 10.1128/AEM.00217-19 PMC6495765

[B59] Lobelle D. Cunliffe M. (2011). Early microbial biofilm formation on marine plastic debris. *Mar. Pollut. Bull.* 62 197–200. 10.1016/j.marpolbul.2010.10.013 21093883

[B60] Martin M. (2011). Cutadapt removes adapter sequences from high-throughput sequencing reads. *EMBnet J.* 17 10–12.

[B61] McDonald D. Clemente J. C. Kuczynski J. Rideout J. R. Stombaugh J. Wendel D. (2012). The Biological observation matrix (BIOM) format or: how I learned to stop worrying and love the ome-ome. *Gigascience* 1:7. 23587224 10.1186/2047-217X-1-7 PMC3626512

[B62] Miao L. Wang P. Hou J. Yao Y. Liu Z. Liu S. (2019). Distinct community structure and microbial functions of biofilms colonizing microplastics. *Sci. Total Environ.* 650 2395–2402. 10.1016/j.scitotenv.2018.09.378 30292995

[B63] Miralles L. Gomez-Agenjo M. Rayon-Viña F. Gyraitë G. Garcia-Vazquez E. (2018). Alert calling in port areas: marine litter as possible secondary dispersal vector for hitchhiking invasive species. *J. Nat. Conserv.* 42 12–18. 10.1016/j.jnc.2018.01.005

[B64] Mor R. Sivan A. (2008). Biofilm formation and partial biodegradation of polystyrene by the actinomycete *Rhodococcus ruber*. *Biodegradation* 19 851–858. 10.1007/s10532-008-9188-0 18401686

[B65] Muthukrishnan T. Al Khaburi M. Abed R. M. M. (2019). Fouling microbial communities on plastics compared with wood and steel: are they substrate- or location-specific? *Microb. Ecol.* 78 361–374. 10.1007/s00248-018-1303-0 30535914

[B66] Oberbeckmann S. Kreikemeyer B. Labrenz M. (2018). Environmental factors support the formation of specific bacterial assemblages on microplastics. *Front. Microbiol.* 8:2709. 10.3389/fmicb.2017.02709 29403454 PMC5785724

[B67] Oberbeckmann S. Loeder M. G. J. Gerdts G. Osborn A. M. (2014). Spatial and seasonal variation in diversity and structure of microbial biofilms on marine plastics in Northern European waters. *FEMS Microbiol. Ecol.* 90 478–492. 10.1111/1574-6941.12409 25109340

[B68] Oberbeckmann S. Osborn A. M. Duhaime M. B. (2016). Microbes on a bottle: substrate, season and geography influence community composition of microbes colonizing marine plastic debris. *PLoS One* 11:e0159289. 10.1371/journal.pone.0159289 27487037 PMC4972250

[B69] Paço A. Duarte K. Da Costa J. P. Santos P. S. M. Pereira R. Pereira M. E. (2017). Biodegradation of polyethylene microplastics by the marine fungus *Zalerion maritimum*. *Sci. Total Environ.* 586 10–15. 10.1016/j.scitotenv.2017.02.017 28199874

[B70] Palm G. J. Reisky L. Böttcher D. Müller H. Michels E. A. P. Walczak M. C. (2019). Structure of the plastic-degrading Ideonella sakaiensis MHETase bound to a substrate. *Nat. Commun.* 10:1717. 30979881 10.1038/s41467-019-09326-3 PMC6461665

[B71] Parada A. E. Needham D. M. Fuhrman J. A. (2016). Every base matters: assessing small subunit rRNA primers for marine microbiomes with mock communities, time series and global field samples. *Environ. Microbiol.* 18 1403–1414. 10.1111/1462-2920.13023 26271760

[B72] Pedrotti M. L. Petit S. Elineau A. Bruzaud S. Crebassa J.-C. Dumontet B. (2016). Changes in the floating plastic pollution of the mediterranean sea in relation to the distance to land. *PLoS One* 11:e0161581. 10.1371/journal.pone.0161581 27556233 PMC4996504

[B73] Pernthaler A. Pernthaler J. Amann R. (2002a). Fluorescence in situ hybridization and catalyzed reporter deposition for the identification of marine bacteria. *Appl. Environ. Microbiol.* 68 3094–3101. 10.1128/aem.68.6.3094-3101.2002 12039771 PMC123953

[B74] Pernthaler A. Preston C. M. Pernthaler J. Delong E. F. Amann R. (2002b). Comparison of fluorescently labeled oligonucleotide and polynucleotide probes for the detection of pelagic marine bacteria and archaea. *Appl. Environ. Microbiol.* 68:661. 10.1128/aem.68.2.661-667.2002 11823205 PMC126737

[B75] Pinto M. Langer T. M. Hüffer T. Hofmann T. Herndl G. J. (2019). The composition of bacterial communities associated with plastic biofilms differs between different polymers and stages of biofilm succession. *PLoS One* 14:e0217165. 10.1371/journal.pone.0217165 31166981 PMC6550384

[B76] Piperagkas O. Papageorgiou N. Karakassis I. (2019). Qualitative and quantitative assessment of microplastics in three sandy Mediterranean beaches, including different methodological approaches. *Estuar. Coast. Shelf Sci.* 219 169–175. 10.1016/j.ecss.2019.02.016

[B77] Prata J. C. Da Costa J. P. Duarte A. C. Rocha-Santos T. (2019). Methods for sampling and detection of microplastics in water and sediment: a critical review. *TrAC Trends Anal. Chem.* 110 150–159. 10.1016/j.trac.2018.10.029

[B78] Prince R. C. Gramain A. Mcgenity T. J. (2010). “Prokaryotic hydrocarbon degraders,” in *Handbook of Hydrocarbon and Lipid Microbiology*, ed. Timmis K. N. (Berlin: Springer Berlin Heidelberg), 1669–1692. 10.1007/978-3-540-77587-4_118

[B79] Probandt D. Eickhorst T. Ellrott A. Amann R. Knittel K. (2018). Microbial life on a sand grain: from bulk sediment to single grains. *ISME J.* 12 623–633. 10.1038/ismej.2017.197 29192905 PMC5776476

[B80] Qin W. Heal K. R. Ramdasi R. Kobelt J. N. Martens-Habbena W. Bertagnolli A. D. (2017). *Nitrosopumilus maritimus* gen. nov., sp. nov., *Nitrosopumilus cobalaminigenes* sp. nov., *Nitrosopumilus oxyclinae* sp. nov., and *Nitrosopumilus ureiphilus* sp. nov., four marine ammonia-oxidizing archaea of the phylum Thaumarchaeota. *Int. J. Syst. Evol. Microbiol.* 67 5067–5079. 10.1099/ijsem.0.002416 29034851

[B81] Quast C. Pruesse E. Yilmaz P. Gerken J. Schweer T. Yarza P. (2013). The SILVA ribosomal RNA gene database project: improved data processing and web-based tools. *Nucleic Acids Res.* 41 D590–D596. 23193283 10.1093/nar/gks1219 PMC3531112

[B82] Raghul S. S. Bhat S. G. Chandrasekaran M. Francis V. Thachil E. T. (2014). Biodegradation of polyvinyl alcohol-low linear density polyethylene-blended plastic film by consortium of marine benthic vibrios. *Int. J. Environ. Sci. Technol.* 11 1827–1834. 10.1007/s13762-013-0335-8

[B83] Rognes T. Flouri T. Nichols B. Quince C. Mahé F. (2016). VSEARCH: a versatile open source tool for metagenomics. *PeerJ* 4 e2584. 10.7717/peerj.2584 27781170 PMC5075697

[B84] Romera-Castillo C. Pinto M. Langer T. M. Álvarez-Salgado X. A. Herndl G. J. (2018). Dissolved organic carbon leaching from plastics stimulates microbial activity in the ocean. *Nat. Commun.* 9:1430. 29651045 10.1038/s41467-018-03798-5 PMC5897397

[B85] Sangeetha Devi R. Rajesh Kannan V. Nivas D. Kannan K. Chandru S. Robert Antony A. (2015). Biodegradation of HDPE by *Aspergillus* spp. from marine ecosystem of Gulf of Mannar, India. *Mar. Pollut. Bull.* 96 32–40. 10.1016/j.marpolbul.2015.05.050 26006776

[B86] Sarkhel R. Sengupta S. Das P. Bhowal A. (2019). Comparative biodegradation study of polymer from plastic bottle waste using novel isolated bacteria and fungi from marine source. *J. Polymer Res.* 27:16.

[B87] Savinelli B. Vega Fernández T. Galasso N. M. D’anna G. Pipitone C. Prada F. (2020). Microplastics impair the feeding performance of a Mediterranean habitat-forming coral. *Mar. Environ. Res.* 155:104887. 10.1016/j.marenvres.2020.104887 32072989

[B88] Schlundt C. Mark Welch J. L. Knochel A. M. Zettler E. R. Amaral-Zettler L. A. (2020). Spatial structure in the “Plastisphere”: molecular resources for imaging microscopic communities on plastic marine debris. *Mol. Ecol. Resour.* 20 620–634. 10.1111/1755-0998.13119 31782619 PMC7318237

[B89] Siboni N. Lidor M. Kramarsky-Winter E. Kushmaro A. (2007). Conditioning film and initial biofilm formation on ceramics tiles in the marine environment. *FEMS Microbiol. Lett.* 274 24–29. 10.1111/j.1574-6968.2007.00809.x 17578524

[B90] Silva M. M. Maldonado G. C. Castro R. O. De Sá Felizardo J. Cardoso R. P. Anjos R. M. D. (2019) Dispersal of potentially pathogenic bacteria by plastic debris in Guanabara Bay. RJ, Brazil. *Mar. Pollut. Bull.* 141 561–568. 10.1016/j.marpolbul.2019.02.064 30955768

[B91] Suaria G. Avio C. G. Mineo A. Lattin G. L. Magaldi M. G. Belmonte G. (2016). The mediterranean plastic soup: synthetic polymers in mediterranean surface waters. *Sci. Rep.* 6:37551. 27876837 10.1038/srep37551 PMC5120331

[B92] Tanasupawat S. Takehana T. Yoshida S. Hiraga K. Oda K. (2016). Ideonella sakaiensis sp. nov., isolated from a microbial consortium that degrades poly(ethylene terephthalate). *Int. J. Syst. Evol. Microbiol.* 66 2813–2818. 10.1099/ijsem.0.001058 27045688

[B93] Thompson H. F. Summers S. Yuecel R. Gutierrez T. (2020). Hydrocarbon-degrading bacteria found tightly associated with the 50-70 μm Cell-size population of eukaryotic phytoplankton in surface waters of a Northeast Atlantic Region. *Microorganisms* 8:1955. 10.3390/microorganisms8121955 33317100 PMC7763645

[B94] Tu C. Chen T. Zhou Q. Liu Y. Wei J. Waniek J. J. (2020). Biofilm formation and its influences on the properties of microplastics as affected by exposure time and depth in the seawater. *Sci. Total Environ.* 734:139237. 10.1016/j.scitotenv.2020.139237 32450399

[B95] van Sebille E. Wilcox C. Lebreton L. Maximenko N. Hardesty B. D. Van Franeker J. A. (2015). A global inventory of small floating plastic debris. *Environ. Res. Lett.* 10:124006. 10.1088/1748-9326/10/12/124006

[B96] Vaksmaa A. Hernando Moreales V. Zeghal E. Niemann H. (2021). Microbial degradation of marine plastics: current state and future prospects. *Biotec. Sustain. Environ.* 978-981-16-1954-0. 10.1007/978-981-16-1955-7_5

[B97] Vianello A. Boldrin A. Guerriero P. Moschino V. Rella R. Sturaro A. (2013). Microplastic particles in sediments of Lagoon of Venice, Italy: first observations on occurrence, spatial patterns and identification. *Estuar. Coast. Shelf Sci.* 130 54–61. 10.1016/j.ecss.2013.03.022

[B98] Wayman C. Niemann H. (2021). The fate of plastic in the ocean environment – a minireview. *Environ. Sci. Process. Impact.* 23 198–212. 10.1039/d0em00446d 33475108

[B99] Woodall L. C. Jungblut A. D. Hopkins K. Hall A. Robinson L. F. Gwinnett C. (2018). Deep-sea anthropogenic macrodebris harbours rich and diverse communities of bacteria and archaea. *PLoS One* 13:e0206220. 10.1371/journal.pone.0206220 30485275 PMC6261660

[B100] Wright R. J. Erni-Cassola G. Zadjelovic V. Latva M. Christie-Oleza J. A. (2020). Marine plastic debris: a new surface for microbial colonization. *Environ. Sci. Technol.* 54 11657–11672. 10.1021/acs.est.0c02305 32886491

[B101] Xanthos D. Walker T. R. (2017). International policies to reduce plastic marine pollution from single-use plastics (plastic bags and microbeads): a review. *Mar. Pollut. Bull.* 118 17–26. 10.1016/j.marpolbul.2017.02.048 28238328

[B102] Xu X. Wang S. Gao F. Li J. Zheng L. Sun C. (2019). Marine microplastic-associated bacterial community succession in response to geography, exposure time, and plastic type in China’s coastal seawaters. *Mar. Pollut. Bull.* 145 278–286. 10.1016/j.marpolbul.2019.05.036 31590788

[B103] Yakimov M. M. Timmis K. N. Golyshin P. N. (2007). Obligate oil-degrading marine bacteria. *Curr. Opin. Biotechnol.* 18 257–266. 10.1016/j.copbio.2007.04.006 17493798

[B104] Yoshida S. Hiraga K. Takehana T. Taniguchi I. Yamaji H. Maeda Y. (2016). A bacterium that degrades and assimilates poly(ethylene terephthalate). *Science* 351 1196–1199. 10.1126/science.aad6359 26965627

[B105] Zadjelovic V. Gibson M. I. Dorador C. Christie-Oleza J. A. (2020). Genome of *Alcanivorax* sp. 24: a hydrocarbon degrading bacterium isolated from marine plastic debris. *Mar. Genomics* 49:100686. 10.1016/j.margen.2019.05.001

[B106] Zettler E. R. Mincer T. J. Amaral-Zettler L. A. (2013). Life in the “Plastisphere”: microbial communities on plastic marine debris. *Environ. Sci. Technol.* 47 7137–7146. 10.1021/es401288x 23745679

[B107] Zhang C. L. Xie W. Martin-Cuadrado A.-B. Rodriguez-Valera F. (2015). Marine group II Archaea, potentially important players in the global ocean carbon cycle. *Front. Microbiol.* 6:1108. 10.3389/fmicb.2015.01108 26528260 PMC4602124

[B108] Zhang J. Gao D. Li Q. Zhao Y. Li L. Lin H. (2020). Biodegradation of polyethylene microplastic particles by the fungus *Aspergillus flavus* from the guts of wax moth *Galleria mellonella*. *Sci. Total Environ.* 704:135931. 10.1016/j.scitotenv.2019.135931 31830656

[B109] Zhang J. Kobert K. Flouri T. Stamatakis A. (2014). PEAR: a fast and accurate Illumina Paired-End reAd mergeR. *Bioinformatics* 30 614–620. 10.1093/bioinformatics/btt593 24142950 PMC3933873

[B110] Zhao S. Zettler E. R. Amaral-Zettler L. A. Mincer T. J. (2020). Microbial carrying capacity and carbon biomass of plastic marine debris. *ISME J.* 15 67–77. 10.1038/s41396-020-00756-2 32879460 PMC7852875

