# Supplementary material for: Microbial Communities on Plastic Polymers in the Mediterranean Sea
Source: Front Microbiol. 2021 Jun 16;12:673553. doi: 10.3389/fmicb.2021.673553 (PMC8243005; doi:10.3389/fmicb.2021.673553)
Supplement: Supplementary Data Sheet 1 — Report of the amplicon sequencing data analysis with Cascabel. [file Data_Sheet_1.PDF]

snakemake report

Amplicon Analysis Report for Library: NIOZ140

CASCABEL is designed to run amplicon sequence analysis across single or multiple read libraries.

The objective of this pipeline is to create different output files which allow the user to explore data in a simple and meaningful way, as well as facilitate downstream analysis, based on the generated output files.

Another aim of **CASCABEL** is also to encourage the documentation process, by creating this report in order to assure data analysis reproducibility.

Following you can see all the steps that were taken in order to get the final results of the pipeline.

Raw Data

The raw data for this library can be found at:

- FW raw reads: Elba16S_pipeline/samples/NIOZ140/rawdata/fw.fastq

- RV raw reads: Elba16S_pipeline/samples/NIOZ140/rawdata/rv.fastq

Number of total reads: 23604452.0

Quality Control

Evaluate quality on raw reads.

Tool: [FastQC]

Version: **FastQC v0.11.7**

Command:

```
fastqc Elba16S_pipeline/samples/NIOZ140/rawdata/fw.fastq Elba16S_pipeline/samples/NIOZ140/rawdata/rv.fastq --extract -o Elba16S_pipeline/samples/NIOZ140/qc/
```

You can follow the links below, in order to see the complete FastQC report:

- FastQC for sample NIOZ140_1: FQ1

- FastQC for sample NIOZ140_2: FQ2

Benchmark info:

s	max_rss	max_vms	max_uss	max_pss	io_in	io_out	mean_load
1039.11	316.36	3914.60	314.09	314.30	11120.93	3.50	0.00

Read pairing

Align paired end reads and merge them into one single sequence in case they overlap.

Tool: [PEAR]

version: **PEAR v0.9.10 [May 30, 2016] - [+bzlib +zlib]**

Command:

```
pear -f Elba16S_pipeline/samples/NIOZ140/rawdata/fw.fastq -r Elba16S_pipeline/samples/NIOZ140/rawdata/rv.fastq -t 50 -v 10 -j 10 -p 0.05 -o Elba16S_pipeline/runs/elba_run_primer_out/NIOZ140_data/peared/seqs > Elba16S_pipeline/runs/elba_run_primer_out/NIOZ140_data/peared/seqs.assembled.fastq
```

Output files:

- Merged reads: Elba16S_pipeline/runs/elba_run_primer_out/NIOZ140_data/peared/seqs.assembled.fastq

- Log file: Elba16S_pipeline/runs/elba_run_primer_out/NIOZ140_data/peared/pear.log

Number of peared reads: 23315749.0 = 98.78%

Benchmark info:

s	max_rss	max_vms	max_uss	max_pss	io_in	io_out	mean_load
3294.28	189.73	1036.18	187.73	187.78	0.69	20701.23	0.00

Extract barcodes

Extract the barcodes used to identify individual samples.

Tool: [QIIME] - extract_barcodes.py

Version: **extract_barcodes.py 1.9.1**

Command:

```
extract_barcodes.py -f Elba16S_pipeline/runs/elba_run_primer_out/NIOZ140_data/peared/seqs.assembled.fastq -c
barcode_paired_stitched --bc1_len 12 --bc2_len 12 -o Elba16S_pipeline/runs/elba_run_primer_out/NIOZ140_data/barcodes/
```

Output files:

- Fastq file with barcodes: Elba16S_pipeline/runs/elba_run_primer_out/NIOZ140_data/barcodes/barcodes.fastq

- Fastq file with the reads: Elba16S_pipeline/runs/elba_run_primer_out/NIOZ140_data/barcodes/reads.fastq

Benchmark info:

s	max_rss	max_vms	max_uss	max_pss	io_in	io_out	mean_load
627.15	120.26	5574.37	92.50	100.89	20238.26	21314.34	0.00

Correct Barcodes

Try to correct the barcode from unassigned reads.

Maximum number of mismatches **2**.

Tool: CASCABEL's R script

Command:

```
Rscript Scripts/errorCorrectBarcodes.R $PWD Elba16S_pipeline/metadata/sampleList_mergedBarcodes_NIOZ140.txt
Elba16S_pipeline/runs/elba_run_primer_out/NIOZ140_data/barcodes/barcodes.fastq 2
```

Output file:

- Barcode corrected file: Elba16S_pipeline/runs/elba_run_primer_out/NIOZ140_data/barcodes/barcodes.fastq_corrected

Benchmark info:

s	max_rss	max_vms	max_uss	max_pss	io_in	io_out	mean_load
3895.77	1154.13	1707.59	1143.99	1145.13	2343.86	2360.46	0.00

Demultiplexing

Library splitting, also known as demultiplexing is carried on several steps.

Split samples from Fastq file

Tool: [QIIME] - split_libraries_fastq.py

version:

Command:

```
split_libraries_fastq.py -m Elba16S_pipeline/metadata/sampleList_mergedBarcodes_NIOZ140.txt -i Elba16S_pipeline/runs/elba_run_primer_out/NIOZ140_data/barcodes/reads.fastq -o Elba16S_pipeline/runs/elba_run_primer_out/NIOZ140_data/splitLibs -b Elba16S_pipeline/runs/elba_run_primer_out/NIOZ140_data/barcodes/barcodes.fastq_corrected -q 20 -r 3 --retain_unassigned_reads --barcode_type 24
```

Benchmark info:

s	max_rss	max_vms	max_uss	max_pss	io_in	io_out	mean_load
1443.48	815.28	5745.60	786.16	793.16	2343.85	11611.82	0.00

Retain assigned reads

Command:

```
cat Elba16S_pipeline/runs/elba_run_primer_out/NIOZ140_data/splitLibs/seqs.fna | grep -P -A1 "(?!>Unass)^>" | sed '/^--$/d' > Elba16S_pipeline/runs/elba_run_primer_out/NIOZ140_data/splitLibs/seqs.assigned.fna
```

Create file with only unassigned reads

Command:

```
cat Elba16S_pipeline/runs/elba_run_primer_out/NIOZ140_data/splitLibs/seqs.fna | grep "^>Unassigned" | sed 's/>Unassigned_[0-9]* /@/g' | sed 's/ .*//' | grep -F -w -A3 -f - Elba16S_pipeline/runs/elba_run_primer_out/NIOZ140_data/peared/seqs.assembled.fastq | sed '/^--$/d' >Elba16S_pipeline/runs/elba_run_primer_out/NIOZ140_data/splitLibs/unassigned.fastq
```

Reverse complement unassigned reads

Tool: [Vsearch]

version: **Rognes T, Flouri T, Nichols B, Quince C, Mahe F (2016)**

Command:

```
vsearch --fastx_revcomp Elba16S_pipeline/runs/elba_run_primer_out/NIOZ140_data/splitLibs/unassigned.fastq --fastqout Elba16S_pipeline/runs/elba_run_primer_out/NIOZ140_data/splitLibs/unassigned.reversed.fastq
```

Barcode extraction for reverse complemented, unassigned reads

Tool: [QIIME] - extract_barcodes.py

Version: **extract_barcodes.py 1.9.1**

Command:

```
extract_barcodes.py -f Elba16S_pipeline/runs/elba_run_primer_out/NIOZ140_data/splitLibs/unassigned.reversed.fastq -c barcode_paired_stitched --bc1_len 12 --bc2_len 12 -o Elba16S_pipeline/runs/elba_run_primer_out/NIOZ140_data/barcodes_unassigned/
```

Correct reverse complemented barcodes

Maximum number of mismatches **2**.

Tool: CASCABEL's R script

Command:

```
Rscript Scripts/errorCorrectBarcodes.R $PWD Elba16S_pipeline/metadata/sampleList_mergedBarcodes_NIOZ140.txt Elba16S_pipeline/runs/elba_run_primer_out/NIOZ140_data/barcodes_unassigned/barcodes.fastq_corrected 2
```

Output file:

- Barcode corrected file: Elba16S_pipeline/runs/elba_run_primer_out/NIOZ140_data/barcodes/barcodes.fastq_corrected

Benchmark info:

s	max_rss	max_vms	max_uss	max_pss	io_in	io_out	mean_load
3103.16	1158.81	1796.82	1159.46	1160.60	1828.30	1828.65	0.00

Split reverse complemented reads

Tool: [QIIME] - extract_barcodes.py

Version: **extract_barcodes.py 1.9.1**

Command:

```
split_libraries_fastq.py -m Elba16S_pipeline/metadata/sampleList_mergedBarcodes_NIOZ140.txt -i
Elba16S_pipeline/runs/elba_run_primer_out/NIOZ140_data/barcodes_unassigned/reads.fastq -o
Elba16S_pipeline/runs/elba_run_primer_out/NIOZ140_data/splitLibsRC -b
Elba16S_pipeline/runs/elba_run_primer_out/NIOZ140_data/barcodes_unassigned/barcodes.fastq_corrected -q 20 -r 3 --
barcode_type 24
```

Benchmark info:

s	max_rss	max_vms	max_uss	max_pss	io_in	io_out	mean_load
1443.48	815.28	5745.60	786.16	793.16	2343.85	11611.82	0.00

Output files:

- FW reads fasta file with new header: Elba16S_pipeline/runs/elba_run_primer_out/NIOZ140_data/splitLibs/seqs.assigned.fna

- Text histogram with the length of the fw reads: Elba16S_pipeline/runs/elba_run_primer_out/NIOZ140_data/splitLibs/histograms.txt

- Log file for the fw reads: Elba16S_pipeline/runs/elba_run_primer_out/NIOZ140_data/splitLibs/split_library_log.txt

- RV reads fasta file with new header: Elba16S_pipeline/runs/elba_run_primer_out/NIOZ140_data/splitLibsRC/seqs.fna

- Text histogram with the length of the rv reads: Elba16S_pipeline/runs/elba_run_primer_out/NIOZ140_data/splitLibsRC/histograms.txt

- Log file for the rv reads: Elba16S_pipeline/runs/elba_run_primer_out/NIOZ140_data/splitLibsRC/split_library_log.txt

Number of reads assigned on FW: 3882450 = 16.65% of the peared reads

Number of reads assigned on RV: 4015672 = 17.22% of the peared reads

Generate single sample fastq files

Create single fastq files per samples (based on the raw data without applying any filtering).

Tool: CASCABEL's Java program

Command:

```
java -cp Scripts DemultiplexQiime --fasta -d Elba16S_pipeline/runs/elba_run_primer_out/NIOZ140_data/seqs_fw_rev_accepted.fna
-o Elba16S_pipeline/runs/elba_run_primer_out/NIOZ140_data/demultiplexed/ -r1
Elba16S_pipeline/samples/NIOZ140/rawdata/fw.fastq.gz -r2 Elba16S_pipeline/samples/NIOZ140/rawdata/fw.fastq.gz
```

The demultiplexed files can be located at:

- demultiplexed directory: Elba16S_pipeline/runs/elba_run_primer_out/NIOZ140_data/demultiplexed/

- summary file: Elba16S_pipeline/runs/elba_run_primer_out/NIOZ140_data/demultiplexed/summary.txt

Benchmark info:

s	max_rss	max_vms	max_uss	max_pss	io_in	io_out	mean_load
7320.60	2187.71	42023.88	2184.70	2184.93	2313.47	1323.70	0.00

Tool: [Cutadapt]

Combine reads

Concatenate forward and reverse reads.

Command:

```
cat Elba16S_pipeline/runs/elba_run_primer_out/NIOZ140_data/splitLibs/seqs.assigned.fna
Elba16S_pipeline/runs/elba_run_primer_out/NIOZ140_data/splitLibsRC/seqs.fna >
Elba16S_pipeline/runs/elba_run_primer_out/NIOZ140_data/seqs_fw_rev_accepted.fna
```

Output files:

- Fasta file with combined reads: Elba16S_pipeline/runs/elba_run_primer_out/NIOZ140_data/seqs_fw_rev_accepted.fna

- Total number of acepted reads: 7898122 = 33.87% of the peared reads or 33.46% of the raw reads.

Benchmark info:

s	max_rss	max_vms	max_uss	max_pss	io_in	io_out	mean_load
6.91	2.96	217.00	1.34	1.36	0.00	4261.15	0.00

Remove sequence primers

Remove the adapters / primers from the reads.

Version: cutadapt v TBD

Command:

```
cutadapt -g GTGYCAGCMGCCGCGGTAA...AAACTYAAAKRAATTGRCGG --match-read-wildcards -O 19-o
Elba16S_pipeline/runs/elba_run_primer_out/NIOZ140_data/seqs_fw_rev_accepted_no_adapters.fna
```

Elba16S_pipeline/runs/elba_run_primer_out/NIOZ140_data/seqs_fw_rev_accepted.fna >
Elba16S_pipeline/runs/elba_run_primer_out/NIOZ140_data/seqs_fw_rev_accepted_no_adapters.log`

Output files:

- Reads without adapters: Elba16S_pipeline/runs/elba_run_primer_out/NIOZ140_data/seqs_fw_rev_accepted_no_adapters.fna

Benchmark info:

s	max_rss	max_vms	max_uss	max_pss	io_in	io_out	mean_load
237.87	13.61	326.65	10.08	10.29	4079.66	3787.61	0.00

Remove too long and too short reads

Remove very short and long reads, with lengths more than some standard deviation below or above the mean to be short or long respectively

- Minimun length expected (shorts): 357

- Maximun length expected (longs): 387

Command:

```
awk '!/^>/ { next } { getline seq } length(seq) > shorts && length(seq) < longs { print $0 "n" seq }'
Elba16S_pipeline/runs/elba_run_primer_out/NIOZ140_data/seqs_fw_rev_accepted.fna >
Elba16S_pipeline/runs/elba_run_primer_out/NIOZ140_data/seqs_fw_rev_filtered.fasta
```

Sequence distribution before remove reads

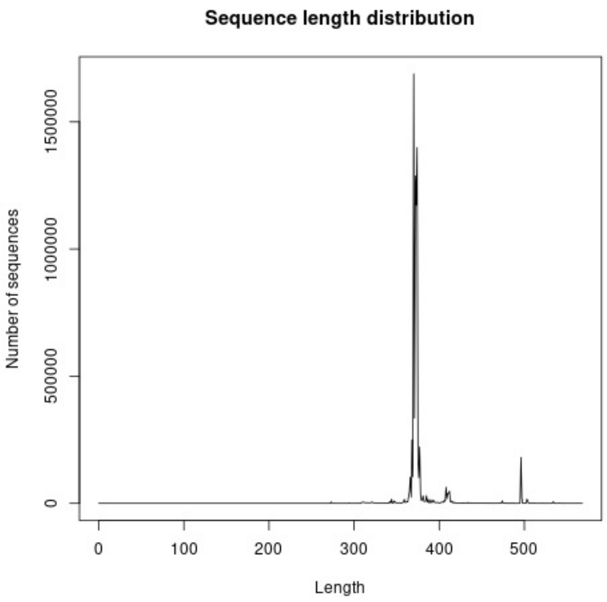

Output file:

- Fasta file with correct sequence length: Elba16S_pipeline/runs/elba_run_primer_out/NIOZ140_data/seqs_fw_rev_filtered.fasta

Total number of reads after length filtering: 7124826

Percentage of reads vs raw reads: 30.18%

Percentage of reads vs demultiplexed reads: 90.21%

Benchmark info:

s	max_rss	max_vms	max_uss	max_pss	io_in	io_out	mean_load
16.05	21.91	471.52	14.33	14.88	0.02	3521.86	0.00

Sample distribution

Sample	Seqs	prc.
NIOZ140.1.1	324346	4.55
NIOZ140.1.2	156472	2.20
NIOZ140.NC	2463	0.03
NIOZ140.1.3	173439	2.43
NIOZ140.1.4	183469	2.58
NIOZ140.1.5	182333	2.56
NIOZ140.1.7	216611	3.04
NIOZ140.1.8	174841	2.45
NIOZ140.1.9	187892	2.64
NIOZ140.1.10	192466	2.70
NIOZ140.1.11	123582	1.73

Sample	Seqs	prc.
NIOZ140.2.1	256969	3.61
NIOZ140.2.2	192918	2.71
NIOZ140.2.3	201367	2.83
NIOZ140.2.4	162368	2.28
NIOZ140.2.5	261124	3.66
NIOZ140.2.6	202616	2.84
NIOZ140.2.7	261507	3.67
NIOZ140.2.8	94571	1.33
NIOZ140.2.9	111162	1.56
NIOZ140.2.10	115332	1.62
NIOZ140.3.1	180300	2.53

Sample	Seqs	prc.
NIOZ140.3.2	372307	5.23
NIOZ140.3.3	110278	1.55
NIOZ140.3.4	216569	3.04
NIOZ140.3.5	363186	5.10
NIOZ140.3.6	237960	3.34
NIOZ140.3.7	56077	0.79
NIOZ140.3.8	41121	0.58
NIOZ140.3.9	368772	5.18
NIOZ140.3.10	731487	10.27
NIOZ140.4.1	220072	3.09
NIOZ140.4.2	114428	1.61

Sample	Seqs	prc.
NIOZ140.4.3	50904	0.71
NIOZ140.4.4	49171	0.69
NIOZ140.4.5	23005	0.32
NIOZ140.4.6	27424	0.38
NIOZ140.4.7	30939	0.43
NIOZ140.4.8	65629	0.92
NIOZ140.4.9	60277	0.85
NIOZ140.4.10	27072	0.38
		ND

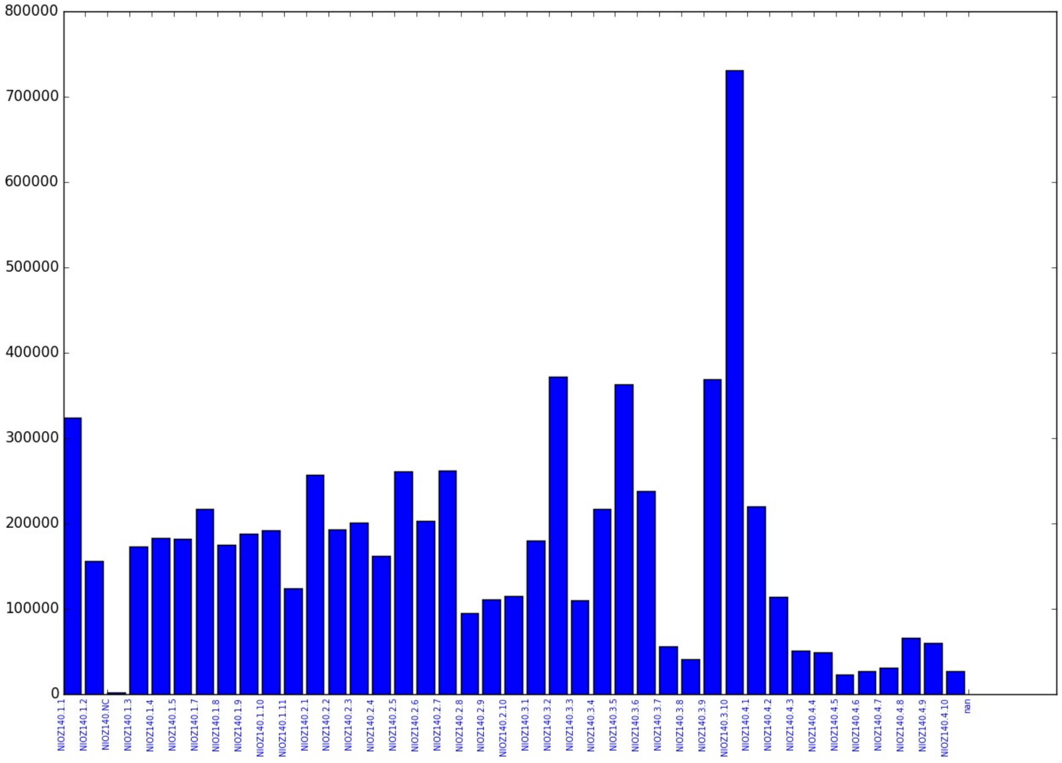

The previous chart shows the number of clean reads per sample. The bars are sorted from left to right, according to the metadata input file.

To see a better detail on the library sample distribution please refere to the file: Elba16S_pipeline/runs/elba_run_primer_out/NIOZ140_data/seqs_fw_rev_filtered.dist.txt

Final counts

Following you can see the final read counts:

File description	Location	Number of reads	Prc(%) vs raw
Raw reads	Elba16S_pipeline/samples/NIOZ140/rawdata/*.fq	23604452.0	100.00%
Assembled reads	Elba16S_pipeline/runs/elba_run_primer_out/NIOZ140_data/peared/seqs.assembled.fastq	23315749.0	98.78%
Demultiplexed reads	Elba16S_pipeline/runs/elba_run_primer_out/NIOZ140_data/seqs_fw_rev_accepted.fna	7898122	33.46%
Adapter removed	Elba16S_pipeline/runs/elba_run_primer_out/NIOZ140_data/seqs_fw_rev_accepted_no_adapters.fna	7898122	33.46%
Length filtered	Elba16S_pipeline/runs/elba_run_primer_out/NIOZ140_data/seqs_fw_rev_filtered.fasta	7124826	30.18%

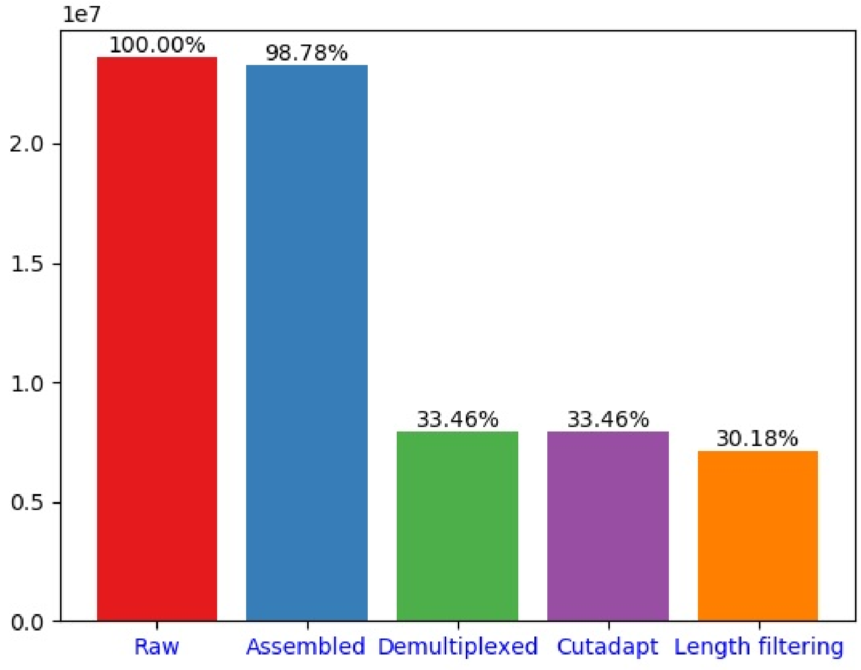

Combine Reads

Merge all the reads of the individual libraries into one single file.

Command:

```
cat Elba16S_pipeline/runs/elba_run_primer_out/NIOZ140_data/seqs_fw_rev_filtered.fasta >
Elba16S_pipeline/runs/elba_run_primer_out/seqs_fw_rev_combined.fasta
```

Output file:

- Merged reads: Elba16S_pipeline/runs/elba_run_primer_out/seqs_fw_rev_filtered.fasta

The total number of reads is: **7124826**

Benchmark info:

s	max_rss	max_vms	max_uss	max_pss	io_in	io_out	mean_load
7.31	21.36	465.03	13.88	14.43	3556.60	3556.60	0.00

Dereplicate reads

Clusterize the reads with an identity threshold of 100%.

Tool: [vsearch]

Version: **Rognes T, Flouri T, Nichols B, Quince C, Mahe F (2016)**

Command:

```
vsearch --derep_fulllength seqs_fw_rev_combined.fasta --output seqs_fw_rev_combined_derep.fasta --uc
seqs_fw_rev_combined_derep.uc --strand both --fasta_width 0 --minuniquesize 1
```

Output files:

- Dereplicated fasta file: Elba16S_pipeline/runs/elba_run_primer_out/derep/seqs_fw_rev_combined_derep.fasta

- Cluster file: Elba16S_pipeline/runs/elba_run_primer_out/derep/seqs_fw_rev_combined_derep.uc

Total number of dereplicated sequences is: **2383291**

Benchmark info:

s	max_rss	max_vms	max_uss	max_pss	io_in	io_out	mean_load
51.27	3369.88	3509.79	3367.57	3367.63	3556.59	1235.50	0.00

Cluster OTUs

Assigns similar sequences to operational taxonomic units, or OTUs, by clustering sequences based on a user-defined similarity threshold.

Tool: [QIIME] - pick_otus.py

Version: **pick_otus.py 1.9.1**

Method: [uclust]

Identity: 0.97

Command:

```
pick_otus.py -m uclust -i Elba16S_pipeline/runs/elba_run_primer_out/seqs_fw_rev_filtered.fasta -o
Elba16S_pipeline/samples/elba_run_primer_out/otu/ -s 0.97
```

Output files:

- OTU List: Elba16S_pipeline/runs/elba_run_primer_out/otu/seqs_fw_rev_filtered_otus.txt

- Log file: Elba16S_pipeline/runs/elba_run_primer_out/otu/seqs_fw_rev_filtered_otus.log

The total number of different OTUS is: **882545**

Benchmark info:

s	max_rss	max_vms	max_uss	max_pss	io_in	io_out	mean_load
14715.41	5522.50	11211.01	5493.58	5498.57	898.04	2038.85	0.00

Pick representatives

Pick a single representative sequence for each OTU.

Tool: [QIIME] - pick_rep_set.py

Version: **pick_rep_set.py 1.9.1**

Method: longest

Command:

```
pick_rep_set.py -m longest -i Elba16S_pipeline/runs/elba_run_primer_out/otu/seqs_fw_rev_filtered_otus.txt -f
Elba16S_pipeline/samples/elba_run_primer_out/seqs_fw_rev_filtered.fasta -o
Elba16S_pipeline/samples/elba_run_primer_out/otu/representative_seq_set.fasta --log_fp
Elba16S_pipeline/samples/elba_run_primer_out/otu/representative_seq_set.log
```

Output file:

- Fasta file with representative sequences: Elba16S_pipeline/runs/elba_run_primer_out/otu/representative_seq_set.fasta

Benchmark info:

s	max_rss	max_vms	max_uss	max_pss	io_in	io_out	mean_load
91.73	4555.76	9415.77	4528.19	4533.18	3614.84	0.02	0.00

Assign taxonomy

Given a set of sequences, assign the taxonomy of each sequence.

Tool: [vsearch]

Version: **Rognes T, Flouri T, Nichols B, Quince C, Mahe F (2016)**

Reference fasta file: /export/data01/databases/silva/qiime/SILVA_132_QIIME_release/rep_set/rep_set_all/99/silva132_99.fna

Taxonomy mapping file: /export/data01/databases/silva/qiime/SILVA_132_QIIME_release/taxonomy/taxonomy_all/99/taxonomy_7_levels.txt

Command:

```
vsearch--usearch_global Elba16S_pipeline/runs/elba_run_primer_out/otu/representative_seq_set.fasta --db
/export/data01/databases/silva/qiime/SILVA_132_QIIME_release/rep_set/rep_set_all/99/silva132_99.fna --dbmask none --qmask
none --rowlen 0 --id 0.7 --iddef 2 --userfields query+id2+target --maxaccepts 3 --threads 10 --top_hits_only --maxrejects 32 --
output_no_hits --userout representative_seq_set_tax_vsearch.out
```

After vsearch assignation, **results were mapped to their LCA using stampa_merge.py** script

The percentage of successfully assigned OTUs is: **99.25%**

Output file:

- OTU taxonomy assignation: Elba16S_pipeline/runs/elba_run_primer_out/otu/taxonomy_vsearch/representative_seq_set_tax_assignments.txt

Benchmark info:

s	max_rss	max_vms	max_uss	max_pss	io_in	io_out	mean_load
1514.57	2319.46	3092.91	2317.09	2317.12	343.47	38.91	0.00

Make OTU table

Tabulates the number of times an OTU is found in each sample, and adds the taxonomic predictions for each OTU in the last column.

Tool: [QIIME] - make_otu_table.py

Version: **make_otu_table.py 1.9.1**

Command:

```
make_otu_table.py -i Elba16S_pipeline/runs/elba_run_primer_out/otu/taxonomy_vsearch/seqs_fw_rev_filtered_otus.txt -t
Elba16S_pipeline/runs/elba_run_primer_out/otu/taxonomy_vsearch/representative_seq_set_tax_assignments.txt -o
Elba16S_pipeline/runs/elba_run_primer_out/otu/taxonomy_vsearch/otuTable.biom
```

Output file:

- Biom format table: Elba16S_pipeline/runs/elba_run_primer_out/otu/taxonomy_vsearch/otuTable.biom

Benchmark info:

s	max_rss	max_vms	max_uss	max_pss	io_in	io_out	mean_load
49.94	2262.51	7122.00	2234.74	2238.87	304.58	277.82	0.00

Convert OTU table

Convert from the BIOM table format to a human readable format.

Tool: [BIOM]

Version: **biom, version 2.1.6**

Command:

```
biom convert -i Elba16S_pipeline/runs/elba_run_primer_out/otu/taxonomy_vsearch/otuTable.biom -o
Elba16S_pipeline/runs/elba_run_primer_out/otu/taxonomy_vsearch/otuTable.txt --table-type 'OTU table' --header-key taxonomy --
to-tsv
```

Output file:

- TSV format table: Elba16S_pipeline/runs/elba_run_primer_out/otu/taxonomy_vsearch/otuTable.txt

Benchmark info:

s	max_rss	max_vms	max_uss	max_pss	io_in	io_out	mean_load
113.50	2652.16	7368.31	2633.04	2635.80	0.00	0.01	0.00

Summarize Taxa

Summarize information of the representation of taxonomic groups within each sample.

Tool: [QIIME] - summarize_taxa.py

Version: **summarize_taxa.py 1.9.1**

Command:

```
summarize_taxa.py -i Elba16S_pipeline/runs/elba_run_primer_out/otu/taxonomy_vsearch/otuTable.biom --level 2,3,4,5,6,7 -o
Elba16S_pipeline/runs/elba_run_primer_out/otu/taxonomy_vsearch/summary/
```

Output file:

- Taxonomy summarized counts at different taxonomy levels: Elba16S_pipeline/runs/elba_run_primer_out/otu/taxonomy_vsearch/summary/otuTable_L**N**.txt

Where **N** is the taxonomy level. Default configuration produces levels from 2 to 6.

Benchmark info:

s	max_rss	max_vms	max_uss	max_pss	io_in	io_out	mean_load
804.57	2824.17	7683.32	2796.39	2801.38	0.00	4.36	0.00

Filter OTU table

Filter OTUs from an OTU table based on their observed counts or identifier.

Tool: [QIIME] - filter_otus_from_otu_table.py

Version: **filter_otus_from_otu_table.py 1.9.1**

Minimum observation counts: 5

Command:

```
filter_otus_from_otu_table.py -i Elba16S_pipeline/runs/elba_run_primer_out/otu/taxonomy_vsearch/otuTable.biom -o
Elba16S_pipeline/runs/elba_run_primer_out/otu/taxonomy_vsearch/otuTable_noSingletons.biom -n 5
```

Output file:

- Biom table: Elba16S_pipeline/runs/elba_run_primer_out/otu/taxonomy_vsearch/otuTable_noSingletons.biom

Benchmark info:

s	max_rss	max_vms	max_uss	max_pss	io_in	io_out	mean_load
56.79	2604.66	7464.34	2577.09	2581.21	278.04	0.02	0.00

Convert Filtered OTU table

Convert the filtered OTU table from the BIOM table format to a human readable format

Tool: [BIOM]

Version: **biom, version 2.1.6**

Command:

```
biom convert -i Elba16S_pipeline/runs/elba_run_primer_out/otu/taxonomy_vsearch/otuTable_noSingletons.biom -o
Elba16S_pipeline/runs/elba_run_primer_out/otu/taxonomy_vsearch/otuTable_noSingletons.txt --table-type 'OTU table' --header-key
taxonomy --to-tsv
```

Output file:

- TSV format table: Elba16S_pipeline/runs/elba_run_primer_out/otu/taxonomy_vsearch/otuTable_noSingletons.txt

Benchmark info:

s	max_rss	max_vms	max_uss	max_pss	io_in	io_out	mean_load
56.79	2604.66	7464.34	2577.09	2581.21	278.04	0.02	0.00

Filter representative sequences

Remove sequences according to the filtered OTU biom table.

Tool: [QIIME] - filter_fasta.py

Version: **filter_fasta.py 1.9.1**

Command:

```
filter_fasta.py -f Elba16S_pipeline/samples/elba_run_primer_out/otu/representative_seq_set.fasta -o
Elba16S_pipeline/samples/elba_run_primer_out/otu/taxonomy_vsearch/representative_seq_set_noSingletons.fasta -b
Elba16S_pipeline/samples/elba_run_primer_out/otu/otuTable_noSingletons.biom
```

Output file:

- Filtered fasta file: Elba16S_pipeline/samples/elba_run_primer_out/otu/taxonomy_vsearch/representative_seq_set_noSingletons.fasta

Benchmark info:

s	max_rss	max_vms	max_uss	max_pss	io_in	io_out	mean_load
8.58	262.19	5671.54	234.55	238.68	0.00	24.70	0.00

Krona report

Krona allows hierarchical data to be explored with zooming, multi-layered pie charts.

Tool: [Krona]

These charts were created using the OTU table **without** singletons

The report was executed for all the samples.

Each sample is represented on a separated chart (same html report).

You can see the report at the following link:

- Krona report: kreport

Or access the html file at:

- Krona html file: Elba16S_pipeline/runs/elba_run_primer_out/otu/taxonomy_vsearch/krona_report.html

Benchmark info:

s	max_rss	max_vms	max_uss	max_pss	io_in	io_out	mean_load
11.87	60.59	1019.07	52.89	54.11	70.63	50.74	0.00

Final counts

Following the read counts:

File description	Location	#	(%)
Combined clean reads	Elba16S_pipeline/runs/elba_run_primer_out/seqs_fw_rev_combined.fasta	7124826	100%
Dereplicated reads	Elba16S_pipeline/runs/elba_run_primer_out/derep/seqs_fw_rev_combined_derep.fasta	2383291	33.45%
OTU table	Elba16S_pipeline/runs/elba_run_primer_out/otu/seqs_fw_rev_combined_remapped_otus.txt	882545	12.39%
Taxonomy assignation	Elba16S_pipeline/runs/elba_run_primer_out/otu/taxonomy_vsearch/representative_seq_set_tax_assignments.txt	875895	99.25%
OTU table (no singletons: a > 5)	Elba16S_pipeline/runs/elba_run_primer_out/otu/taxonomy_vsearch/otuTable_noSingletons.txt	64548	7.31%
Assigned no singletons	Elba16S_pipeline/runs/elba_run_primer_out/otu/taxonomy_vsearch/otuTable_noSingletons.txt	63198	97.91%

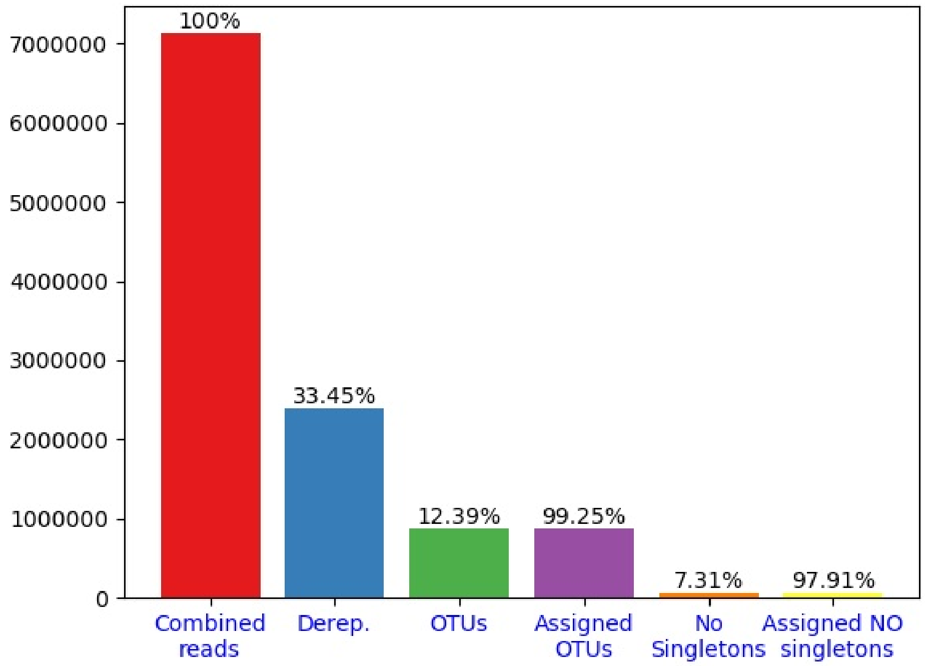

Note:

- Assigned OTUs percentage is the amount of successfully assigned OTUs.

- No singletons percentage is the percentage of no singletons OTUs in reference to the complete OTU table.

- Assigned No singletons is the amount of successfully no singletons assigned OTUs.

References

[FastQC] FastQC v0.11.3. Andrews S. (2010). FastQC: a quality control tool for high throughput sequence data

[PEAR] PEAR: a fast and accurate Illumina Paired-End reAd mergeR. Zhang et al (2014) Bioinformatics 30(5): 614-620 | doi:10.1093/bioinformatics/btt593

[QIIME] QIIME. Caporaso JG, Kuczynski J, Stombaugh J, Bittinger K, Bushman FD, Costello EK, Fierer N, Gonzalez Pena A, Goodrich JK, Gordon JI, Huttley GA, Kelley ST, Knights D, Koenig JE, Ley RE, Lozupone CA, McDonald D, Muegge BD, Pirrung M, Reeder J, Sevinsky JR, Turnbaugh PJ, Walters WA, Widmann J, Yatsunenko T, Zaneveld J, Knight R. 2010. QIIME allows analysis of high-throughput community sequencing data. Nature Methods 7(5): 335-336.

[Cutadapt] Cutadapt v1.15 .Marcel Martin. Cutadapt removes adapter sequences from high-throughput sequencing reads. EMBnet.Journal, 17(1):10-12, May 2011. http://dx.doi.org/10.14806/ej.17.1.200

[Vsearch] Rognes T, Flouri T, Nichols B, Quince C, Mahé F. (2016) VSEARCH: a versatile open source tool for metagenomics. PeerJ 4:e2584. doi: 10.7717/peerj.2584

[BIOM] The Biological Observation Matrix (BIOM) format or: how I learned to stop worrying and love the ome-ome. Daniel McDonald, Jose C. Clemente, Justin Kuczynski, Jai Ram Rideout, Jesse Stombaugh, Doug Wendel, Andreas Wilke, Susan Huse, John Hufnagle, Folker Meyer, Rob Knight, and J. Gregory Caporaso.GigaScience 2012, 1:7. doi:10.1186/2047-217X-1-7

[uclust] Edgar RC. 2010. Search and clustering orders of magnitude faster than BLAST. Bioinformatics 26(19):2460-2461.

Author: J. Engelmann & A. Abdala | 2020-02-19
