# Supplementary figures and images for: Microbial Communities on Plastic Polymers in the Mediterranean Sea
Source: Front Microbiol. 2021 Jun 16;12:673553. doi: 10.3389/fmicb.2021.673553 (PMC8243005; doi:10.3389/fmicb.2021.673553)

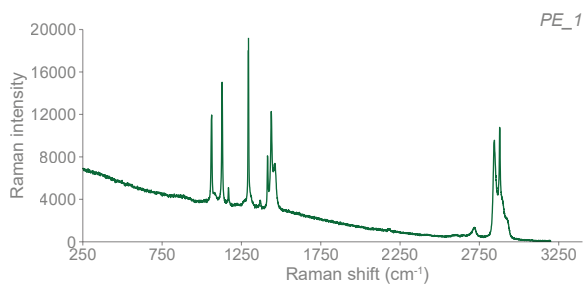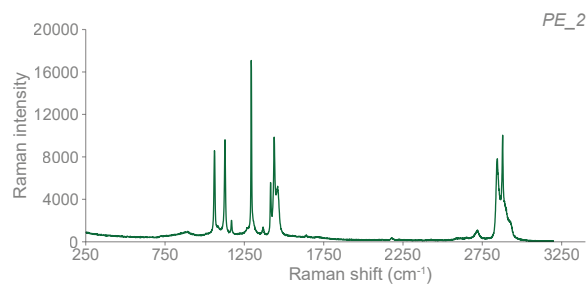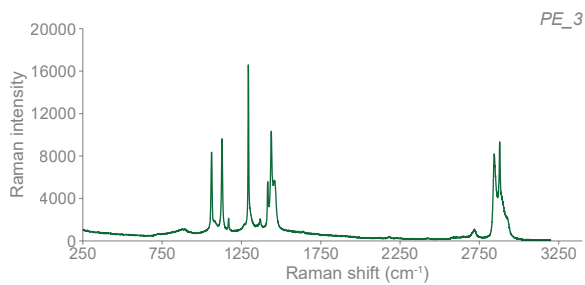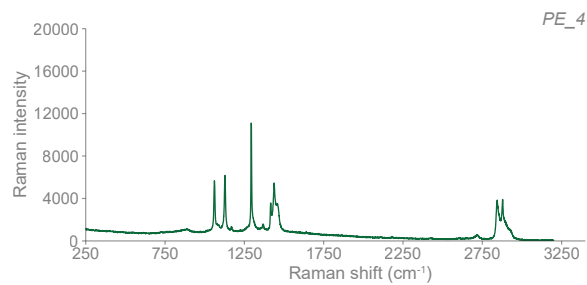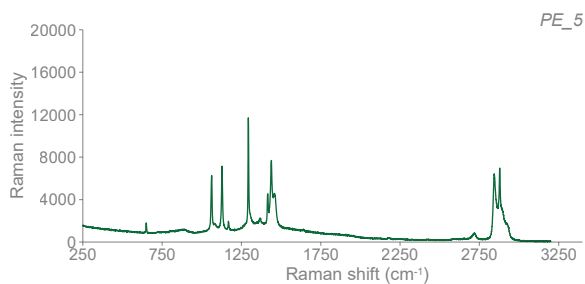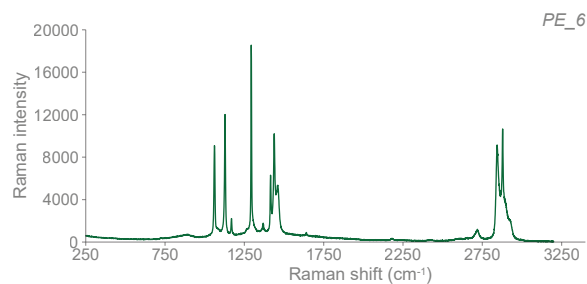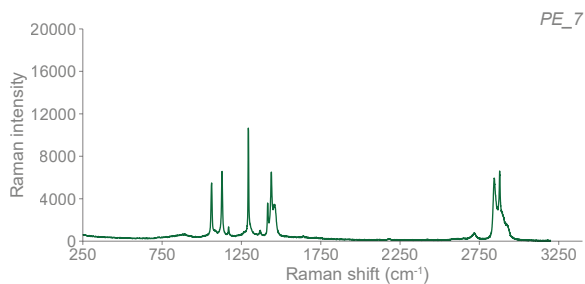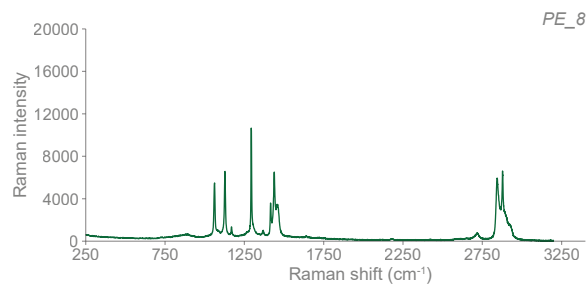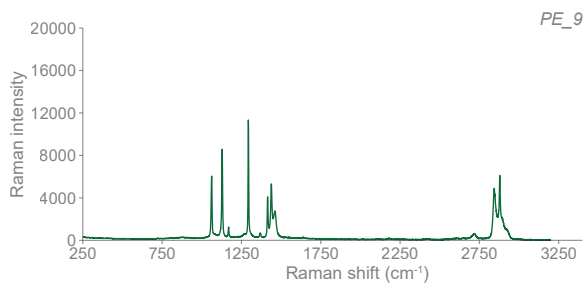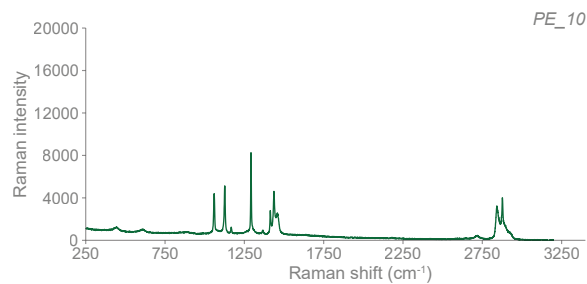

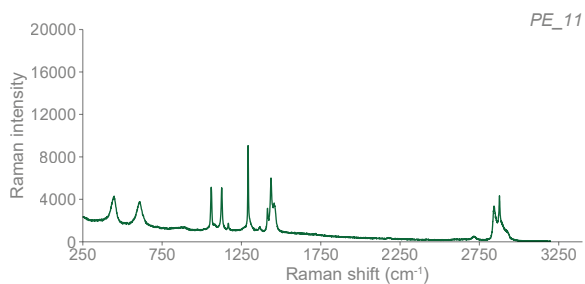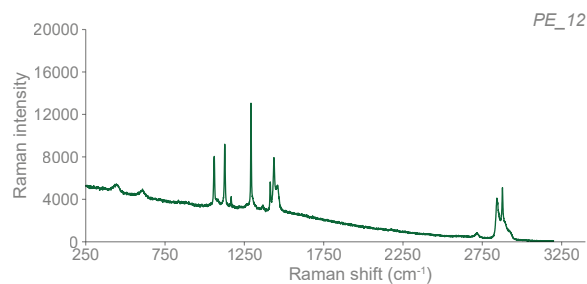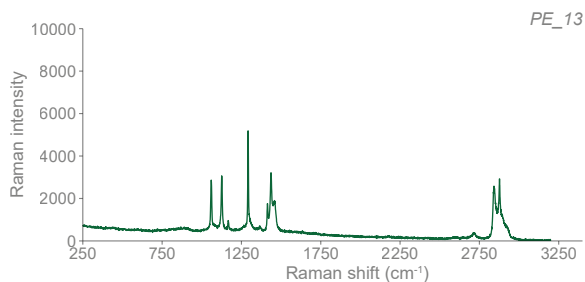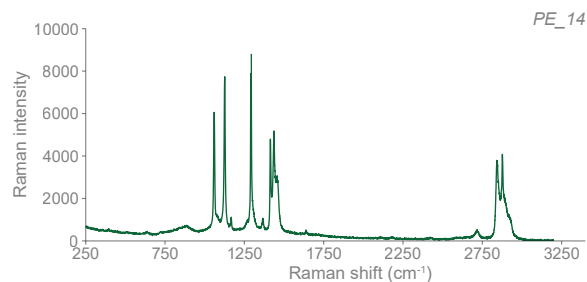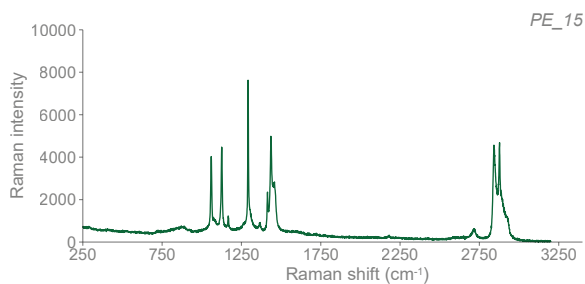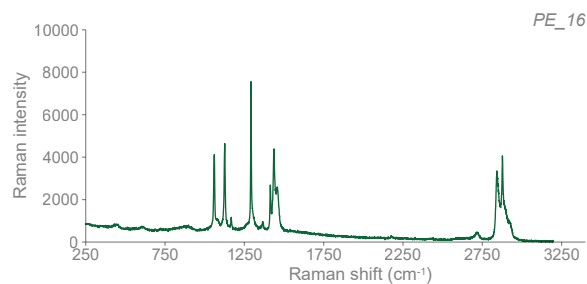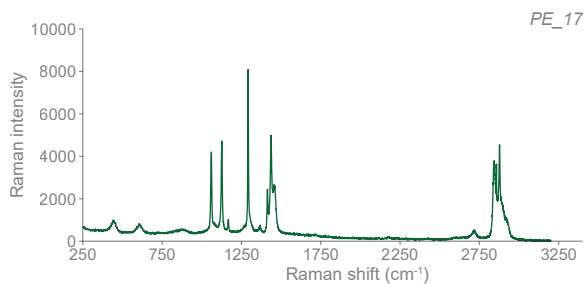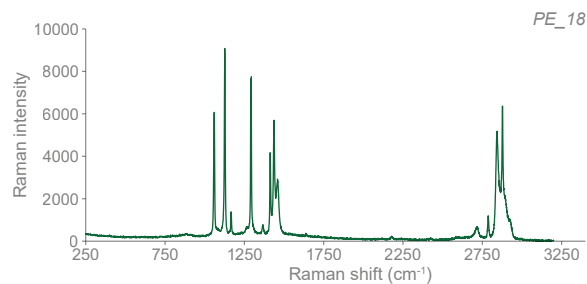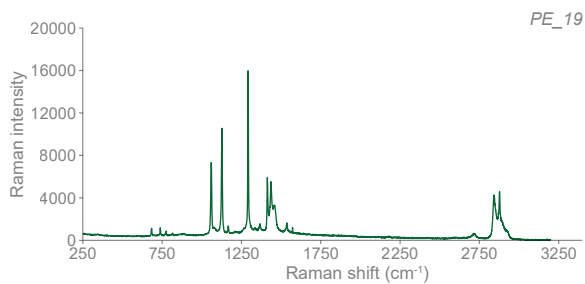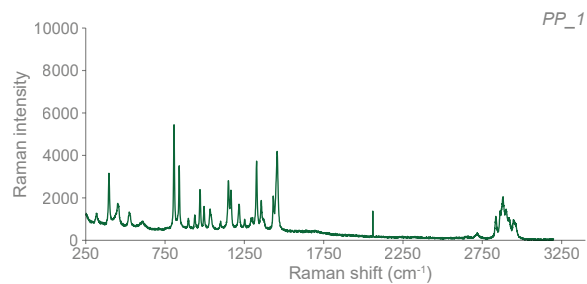

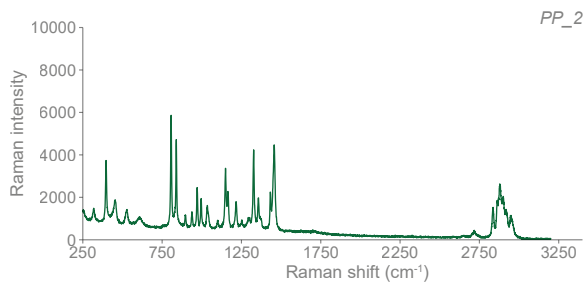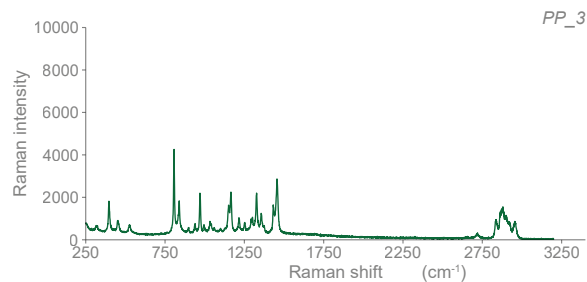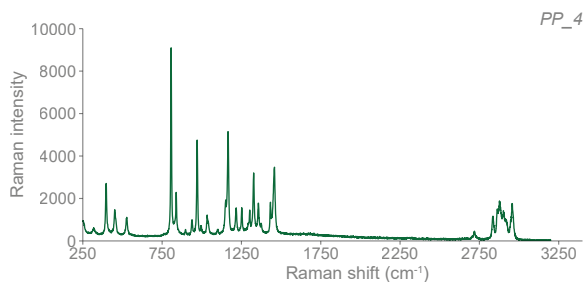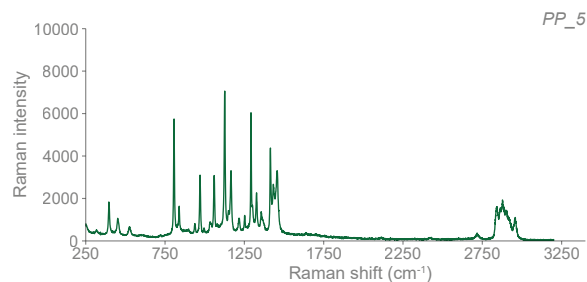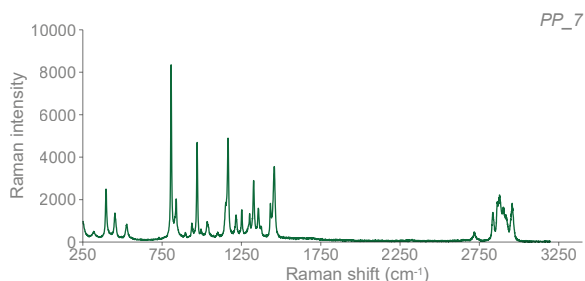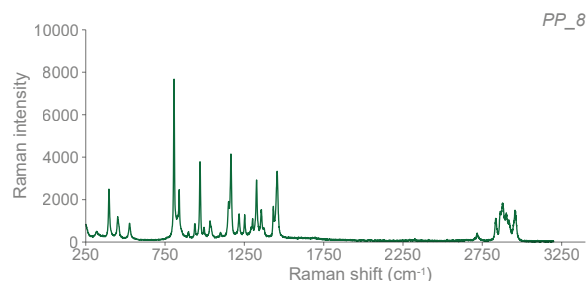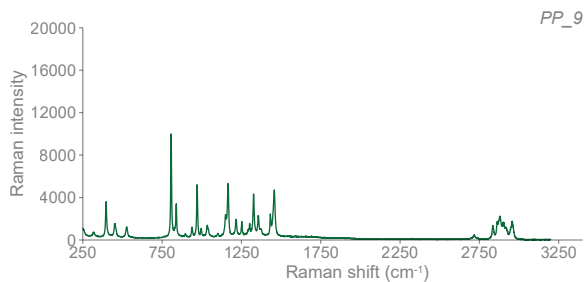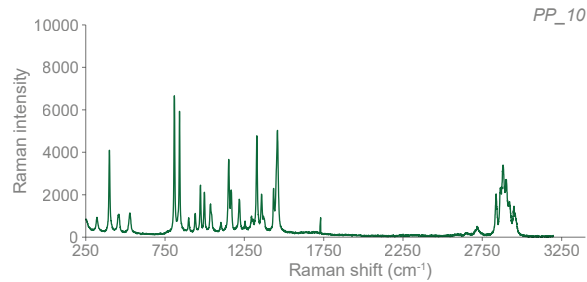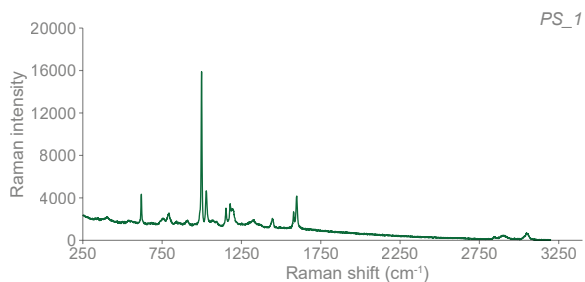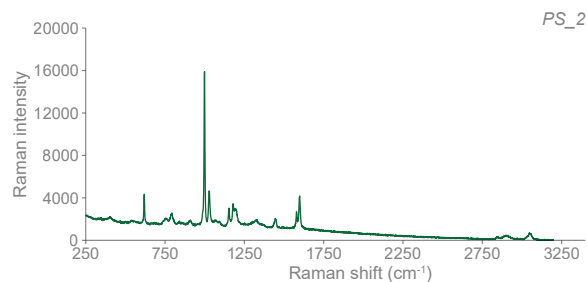

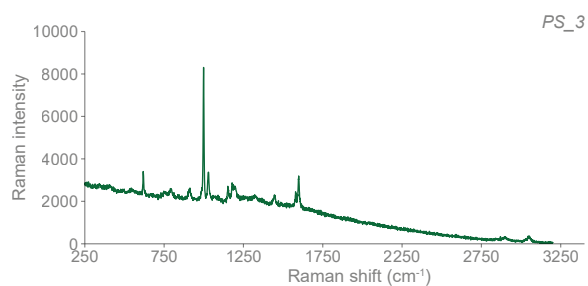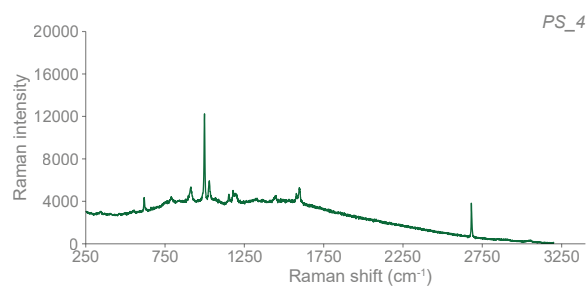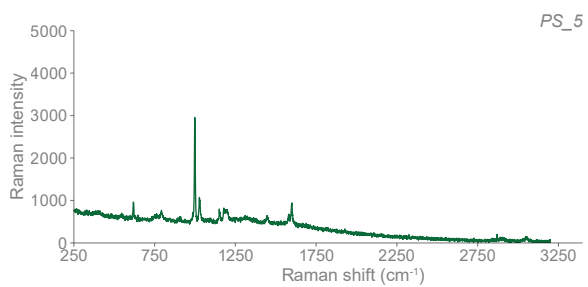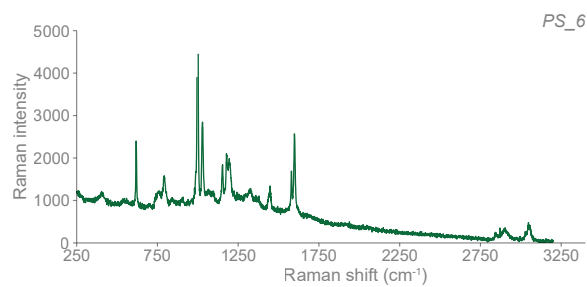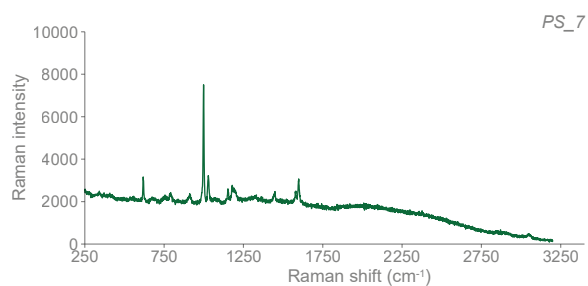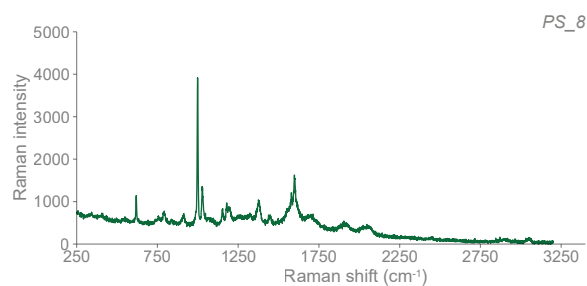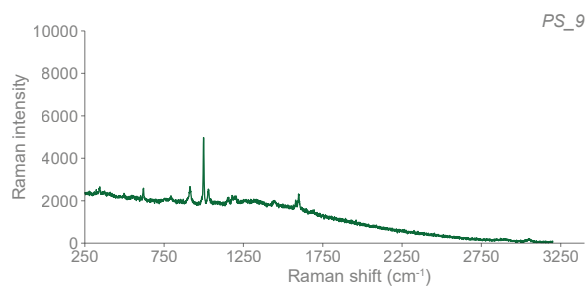

Supplement: Supplementary Data Sheet 2 — Raman Spectra. [file Data_Sheet_2.PDF]

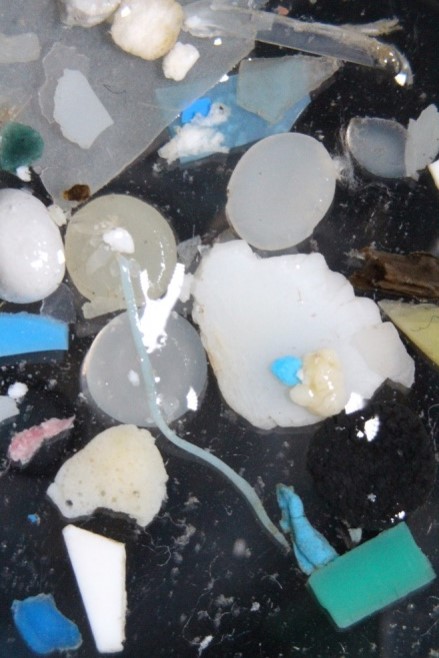

Supplement: Supplementary Figure 1 — Marine plastic debris. [file Image_1.JPEG]
