# Supplementary material for: Microbial Communities on Plastic Polymers in the Mediterranean Sea
Source: Front Microbiol. 2021 Jun 16;12:673553. doi: 10.3389/fmicb.2021.673553 (PMC8243005; doi:10.3389/fmicb.2021.673553)
Supplement: Supplementary Table 1 — Statistical analysis. [file Table_1.DOCX]

**PERMANOVA test results based on Bray-Curtis dissimilarities using abundance data on the OTU and Order level for microbial communities in relation to plastic polymer type**. Df – degrees of freedom; SS – sum of squares, MS- mean sum of squares, Pseudo -F – F statistic, p-values based on permutations, t- t value based on permutations.

| PERMANOVA |  | OTU level |  |  |  |  | Order level | |  |  |  |
| --- | --- | --- | --- | --- | --- | --- | --- | --- | --- | --- | --- |
| Source | df | SS | MS | Pseudo-F | p(perm) | Unique perms | SS | MS | Pseudo-F | P(perm) | Unique perms |
| Gr | 2 | 14389 | 7194.5 | 2.8377 | 1.00^E^-05 | 85432 | 7496 | 3748 | 5.4706 | 0.0001 | 9913 |
| Res | 35 | 88737 | 2535.3 |  |  |  | 23979 | 685.13 |  |  |  |
| Total | 37 | 1.03^E^+05 |  |  |  |  | 31475 |  |  |  |  |

**PAIR-WISE TESTS-Order level**

| Groups | t | P(perm) | Unique perms |
| --- | --- | --- | --- |
| PE, PP | 2.4454 | 0.0002 | 9927 |
| PE, PS | 2.9088 | 0.0001 | 9919 |
| PP, PS | 1.2244 | 0.1413 | 9374 |

**PAIR-WISE TESTS-OTU level**

| Groups | t | P(perm) | Unique perms |
| --- | --- | --- | --- |
| PE, PP | 1.6881 | 0.0002 | 88387 |
| PE, PS | 1.9905 | 1.00E-05 | 88446 |
| PP, PS | 1.1847 | 0.0251 | 55133 |

**PERMDISP-Order level**

Distance-based test for homogeneity of multivariate dispersions

Data type: Similarity

Standardise: Samples by Total

Transform: Square root

Resemblance: S17 Bray-Curtis similarity

Group factor: Group of plastic

Number of permutations: 9999

Number of groups: 3

Number of samples: 38

*DEVIATIONS FROM CENTROID*

F: 5.2158 df1: 2 df2: 35

P(perm): 0.0166

PAIRWISE COMPARISONS

| Groups | t | P(perm) |
| --- | --- | --- |
| PE, PP | 2.8096 | 0.0128 |
| PE, PS | 0.23946 | 0.8331 |
| PP, PS | 3.1329 | 0.0073 |
| PE, PP+PS | 2.1444 | 0.0525 |

*MEANS AND STANDARD ERRORS*

| Group | Size | Average | SE |
| --- | --- | --- | --- |
| PE | 19 | 22.717 | 1.4581 |
| PP | 10 | 29.339 | 1.6849 |
| PS | 9 | 22.148 | 1.5307 |

**PERMDISP-OTU level**

Distance-based test for homogeneity of multivariate dispersions

Data type: Similarity

Standardise: Samples by Total

Transform: Square root

Resemblance: S17 Bray-Curtis similarity

Group factor: Group of plastic

Number of permutations: 9999

Number of groups: 3

Number of samples: 38

*DEVIATIONS FROM CENTROID*

F: 4.1459 df1: 2 df2: 35

P(perm): 0.0594

PAIRWISE COMPARISONS

| Groups | t | P(perm) |
| --- | --- | --- |
| PE, PP | 2.3522 | 0.0479 |
| PE, PS | 0.53066 | 0.6801 |
| PP, PS | 2.7456 | 0.0125 |
| PE, PP+PS | 2.1962 | 0.0434 |

MEANS AND STANDARD ERRORS

| Group | Size | Average | SE |
| --- | --- | --- | --- |
| PE | 19 | 46.622 | 1.4708 |
| PP | 10 | 52.564 | 2.0779 |
| PS | 9 | 45.355 | 1.5208 |

| \| **SIMPER on Order level** \| \| \|  \|  \|  \| \| --- \| --- \| --- \| --- \| --- \| --- \| \|  \|  \|  \|  \|  \|  \| \| *Group PE* \|  \|  \|  \|  \|  \| \| Average similarity: 67.02 \| \| \|  \|  \|  \| \|  \|  \|  \|  \|  \|  \| \| Species \| Av.Abund \| Av.Sim \| Sim/SD \| Contrib% \| Cum.% \| \| Flavobacteriales \| 4.4 \| 5.86 \| 3.16 \| 8.74 \| 8.74 \| \| Rhodobacterales \| 3.04 \| 3.79 \| 2.65 \| 5.66 \| 14.4 \| \| Chitinophagales \| 2.41 \| 3.01 \| 3.04 \| 4.49 \| 18.9 \| \| Caulobacterales \| 2.2 \| 2.59 \| 2.52 \| 3.87 \| 22.77 \| \| Sphingomonadales \| 2.02 \| 2.36 \| 1.73 \| 3.52 \| 26.29 \| \| Alteromonadales \| 1.92 \| 2.2 \| 2.49 \| 3.28 \| 29.57 \| \| Oceanospirillales \| 1.89 \| 2 \| 2.37 \| 2.99 \| 32.55 \| \| Cytophagales \| 1.71 \| 1.98 \| 2.46 \| 2.95 \| 35.51 \| \| Rhizobiales \| 1.72 \| 1.91 \| 2.03 \| 2.85 \| 38.35 \| \| Cellvibrionales \| 1.44 \| 1.58 \| 3.1 \| 2.35 \| 40.71 \| \| Pirellulales \| 1.34 \| 1.51 \| 2.85 \| 2.26 \| 42.97 \| \| Microtrichales \| 1.48 \| 1.41 \| 1.89 \| 2.11 \| 45.07 \| \|  \|  \|  \|  \|  \|  \| \| *Group PP* \|  \|  \|  \|  \|  \| \| Average similarity: 56.54 \| \| \|  \|  \|  \| \|  \|  \|  \|  \|  \|  \| \| Species \| Av.Abund \| Av.Sim \| Sim/SD \| Contrib% \| Cum.% \| \| Flavobacteriales \| 5.61 \| 9.87 \| 2.52 \| 17.45 \| 17.45 \| \| Chitinophagales \| 2.58 \| 4.14 \| 2.33 \| 7.32 \| 24.77 \| \| Rickettsiales \| 2.83 \| 4 \| 1.43 \| 7.08 \| 31.85 \| \| Cytophagales \| 2.53 \| 3.91 \| 2.88 \| 6.91 \| 38.77 \| \| Rhodobacterales \| 2.25 \| 2.97 \| 2.36 \| 5.25 \| 44.01 \| \| Caulobacterales \| 1.68 \| 2.57 \| 3.38 \| 4.55 \| 48.56 \| \| Alteromonadales \| 2.13 \| 2.18 \| 1.2 \| 3.85 \| 52.42 \| \|  \|  \|  \|  \|  \|  \| \| *Group PS* \|  \|  \|  \|  \|  \| \| Average similarity: 66.94 \| \| \|  \|  \|  \| \|  \|  \|  \|  \|  \|  \| \| Species \| Av.Abund \| Av.Sim \| Sim/SD \| Contrib% \| Cum.% \| \| Flavobacteriales \| 5.59 \| 8.41 \| 3.34 \| 12.56 \| 12.56 \| \| Cytophagales \| 3.39 \| 4.92 \| 5.87 \| 7.36 \| 19.92 \| \| Rickettsiales \| 2.88 \| 3.69 \| 2.38 \| 5.51 \| 25.43 \| \| Chitinophagales \| 2.42 \| 3.6 \| 4.69 \| 5.38 \| 30.81 \| \| Rhodobacterales \| 1.56 \| 2.23 \| 3.66 \| 3.34 \| 34.15 \| \| Alteromonadales \| 1.98 \| 2.11 \| 1.42 \| 3.15 \| 37.3 \| \| Caulobacterales \| 1.13 \| 1.73 \| 3.51 \| 2.58 \| 39.87 \| \| Nitrosopumilales \| 2.17 \| 1.71 \| 1.01 \| 2.55 \| 42.42 \| \| Oceanospirillales \| 1.13 \| 1.55 \| 2.62 \| 2.31 \| 44.73 \| | | |  |  |  |
| --- | --- | --- | --- | --- | --- | --- | --- | --- | --- | --- | --- | --- | --- | --- | --- | --- | --- | --- | --- | --- | --- | --- | --- | --- | --- | --- | --- | --- | --- | --- | --- | --- | --- | --- | --- | --- | --- | --- | --- | --- | --- | --- | --- | --- | --- | --- | --- | --- | --- | --- | --- | --- | --- | --- | --- | --- | --- | --- | --- | --- | --- | --- | --- | --- | --- | --- | --- | --- | --- | --- | --- | --- | --- | --- | --- | --- | --- | --- | --- | --- | --- | --- | --- | --- | --- | --- | --- | --- | --- | --- | --- | --- | --- | --- | --- | --- | --- | --- | --- | --- | --- | --- | --- | --- | --- | --- | --- | --- | --- | --- | --- | --- | --- | --- | --- | --- | --- | --- | --- | --- | --- | --- | --- | --- | --- | --- | --- | --- | --- | --- | --- | --- | --- | --- | --- | --- | --- | --- | --- | --- | --- | --- | --- | --- | --- | --- | --- | --- | --- | --- | --- | --- | --- | --- | --- | --- | --- | --- | --- | --- | --- | --- | --- | --- | --- | --- | --- | --- | --- | --- | --- | --- | --- | --- | --- | --- | --- | --- | --- | --- | --- | --- | --- | --- | --- | --- | --- | --- | --- | --- | --- | --- | --- | --- | --- | --- | --- | --- | --- | --- | --- | --- | --- | --- | --- | --- | --- | --- | --- | --- | --- | --- | --- | --- | --- | --- | --- | --- | --- | --- | --- | --- | --- | --- | --- | --- | --- | --- | --- | --- | --- | --- | --- | --- | --- | --- | --- | --- | --- | --- | --- | --- | --- | --- | --- | --- | --- | --- | --- | --- | --- | --- | --- | --- | --- | --- | --- | --- | --- | --- | --- | --- | --- | --- | --- | --- | --- | --- | --- |
|  |  |  |  |  |  |
|  |  |  |  |  |  |
|  | | |  |  |  |
|  |  |  |  |  |  |
|  |  |  |  |  |  |

Non-metric multidimensional scaling (nMDS) of microbial communities on order level based on 16S rRNA gene sequencing from biofilms colonising polyethylene, polystyrene and polypropylene plastic polymers.
